# Sedimentology, petrography, and reservoir quality of the Zarga and Ghazal formations in the Keyi oilfield, Muglad Basin, Sudan

**DOI:** 10.1038/s41598-020-80831-y

**Published:** 2021-01-12

**Authors:** Yousif M. Makeen, Xuanlong Shan, Habeeb A. Ayinla, Ekundayo Joseph Adepehin, Ndip Edwin Ayuk, Nura Abdulmumini Yelwa, Jian Yi, Osman M. A. Elhassan, Daijun Fan

**Affiliations:** 1 College of Earth Sciences, Jilin University, Changchun, 130061 China; 2 Department of Geology, Federal University Lokoja, P. M. B 1154, Lokoja, Nigeria; 3 Department of Geology, National University of Malaysia, Bangi, Malaysia; 4 Pan African University-Life and Earth Science Institute, University of Ibadan, Ibadan, Nigeria; 5 Department of Geology, University of Malaya, 50603 Kuala Lumpur, Malaysia; 6 Harquail School of Earth Sciences, Laurentian University, Sudbury, Canada

**Keywords:** Sedimentology, Core processes

## Abstract

The Zarga and Ghazal formations constitute important reservoirs across the Muglad Basin, Sudan. Nevertheless, the sedimentology and diagenesis of these reservoir intervals have hitherto received insignificant research attention. Detailed understanding of sedimentary facies and diagenesis could enhance geological and geophysical data for better exploration and production and minimize risks. In this study, subsurface reservoir cores representing the Zarga formation (1114.70–1118.50 m and 1118.50–1125.30 m), and the Ghazal formation (91,403.30–1406.83 m) were subjected to sedimentological (lithofacies and grain size), petrographic/mineralogic (thin section, XRD, SEM), and petrophysical (porosity and permeability) analyses to describe their reservoir quality, provenance, and depositional environments. Eight (8) different lithofacies, texturally characterized as moderately to well-sorted, and medium to coarse-grained, sub-feldspathic to feldspathic arenite were distinguished in the cored intervals. Mono-crystalline quartz (19.3–26.2%) predominated over polycrystalline quartz (2.6–13.8%), feldspar (6.6–10.3%), and mica (1.4–7.6%) being the most prominent constituent of the reservoir rocks. Provenance plot indicated the sediments were from a transitional continental provenance setting. The overall vertical sequence, composition, and internal sedimentary structures of the lithofacies suggest a fluvial-to-deltaic depositional environment for the Ghazal formation, while the Zarga formation indicated a dominant deltaic setting. Kaolinite occurs mainly as authigenic mineral, while carbonates quantitatively fluctuate with an insignificant amount of quartz overgrowths in most of the analyzed cores. Integration of XRD, SEM, and thin section analysis highlights that kaolinite, chlorite, illite, and smectite are present as authigenic minerals. Pore-destroying diagenetic processes (e.g. precipitation, cementation, and compaction etc.) generally prevailed over pore-enhancing processes (e.g. dissolution). Point-counted datasets indicate a better reservoir quality for the Ghazal formation (ɸ = 27.7% to 30.7%; K = 9.65 mD to 1196.71 mD) than the Zarga formation (17.9% to 24.5%; K = 1051.09 mD to 1090.45 mD).

## Introduction

**Figure 1:**
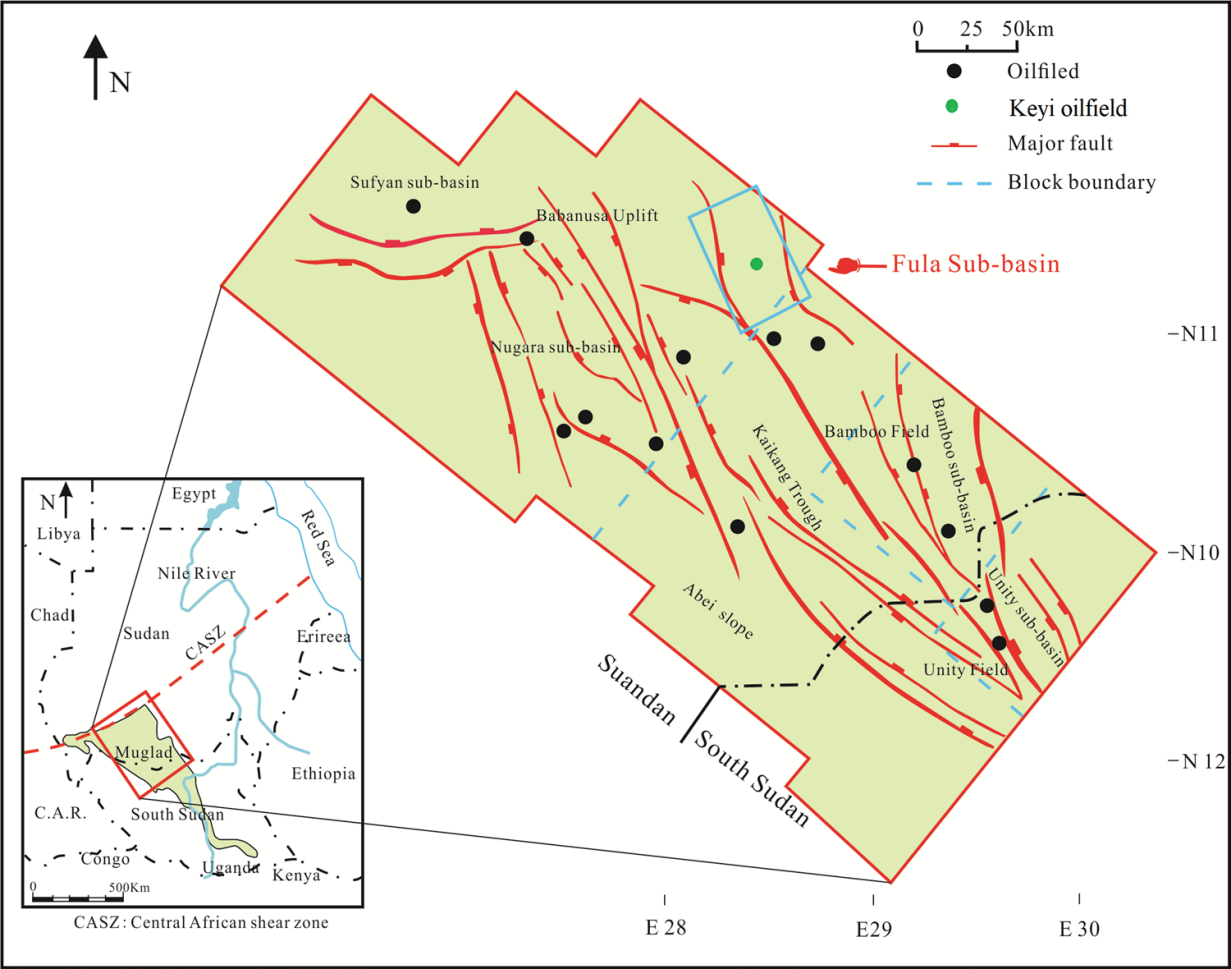
Location of map showing the Keyi oilfield, Northeastern region of the Muglad Basin, Sudan. It also shows the main oilfields discovered, major fault and block boundary (created using CorelDRAW Graphics Suite 2018 v20.0.0.633 https://www.corel.com/cn).

The Muglad Basin is a rift basin and an important hydrocarbon province in northern Africa. It extends about 300 km wide and 1200 km long cutting across the Republic of Sudan and the South Sudan. Hydrocarbon discovery and exploration in the Muglad Basin started in the early 1970s and spans to 1980s and present. The Fula, Heglig and Unity oilfields (Fig. 1) were the first to be discovered during early exploration activities carried out by Chevron Oil^1,2^. Although, the sedimentary thickness at the basin’s depocenter is estimated to be 13.7 km, the maximum drilled thickness in basin is generally < 4.5 km depth penetrating mainly lacustrine and fluvial deposits^1,3–9^.

**Figure 2:**
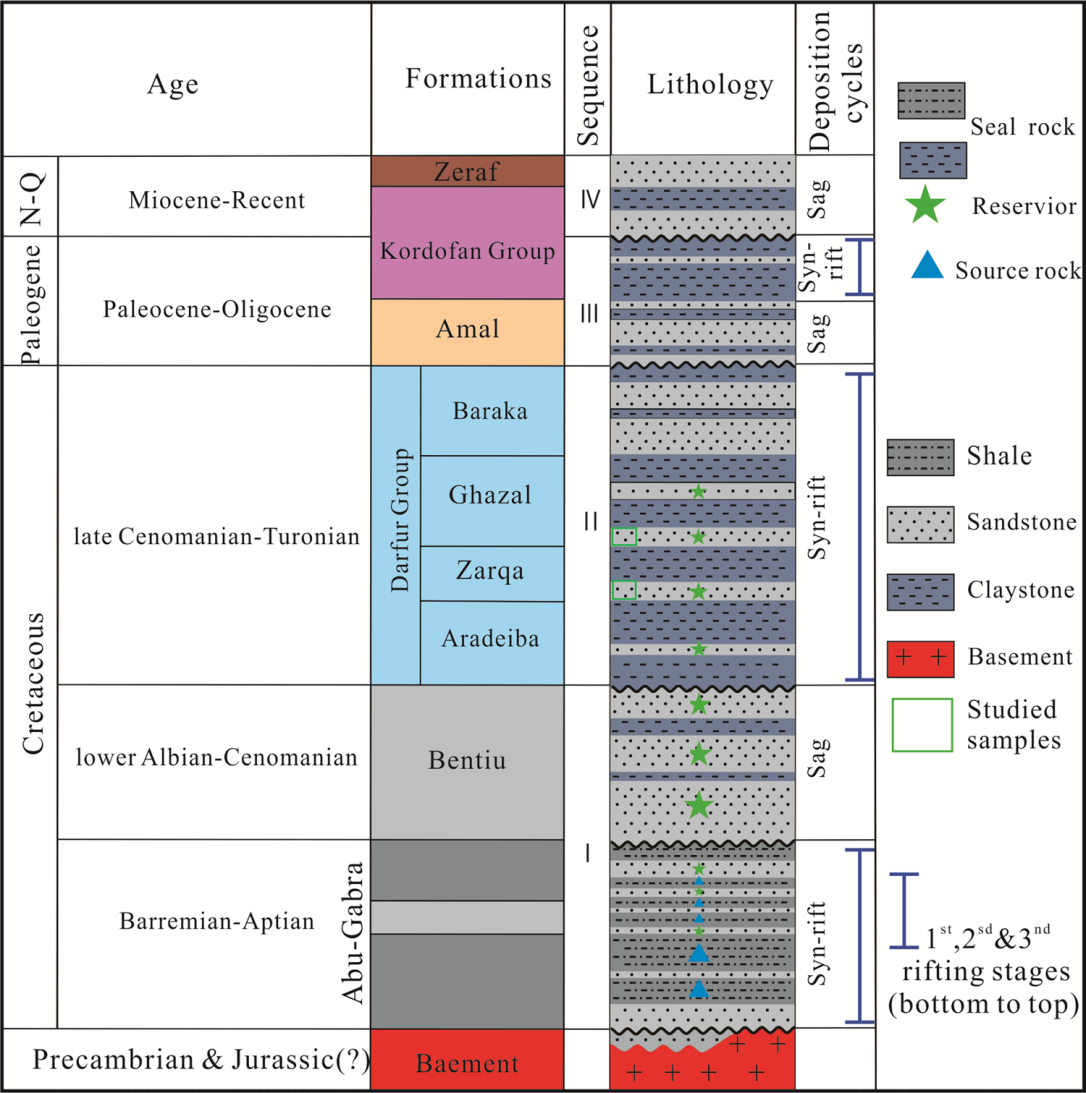
The main stratigraphic column of the Northeastern region of the Muglad Basin. It compares sediments succession from Late Jurassic/Early Cretaceous—Quaternary with four sequences (I–IV) that separated by unconformities.

**Figure 3:**
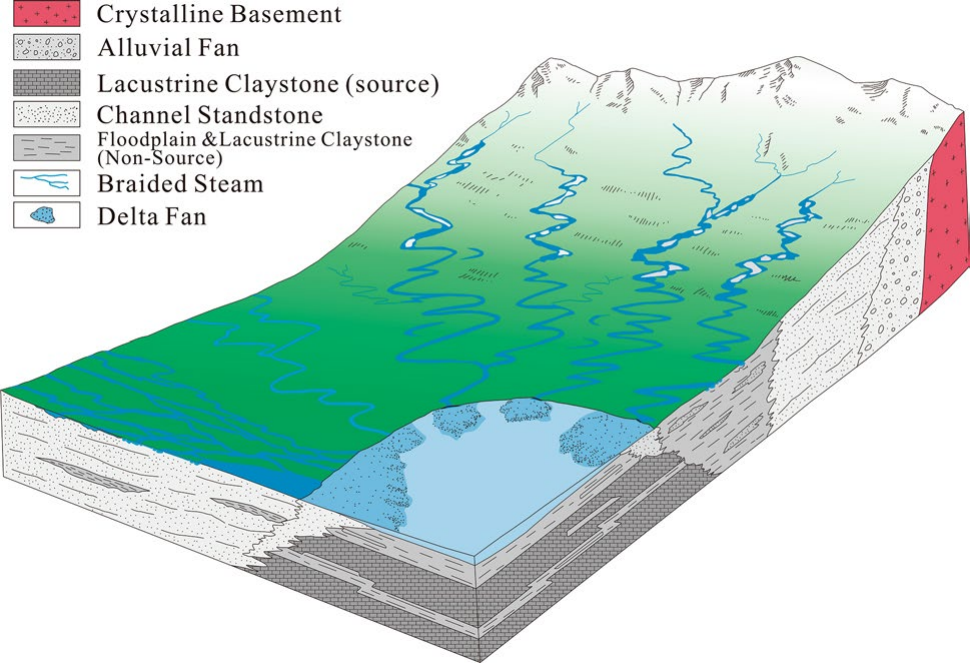
Depositional model showing the environmental effects during the filling of the Muglad Basin (created from information in reference^1^).

Over the years, the Muglad Basin has attracted substantial exploration/research activities being the main hydrocarbon province in the Republic of Sudan and South Sudan. Early exploration activities include; Aerial photography, Landsat images, airborne magnetometer, helicopter supported gravity survey and detail seismic survey carried by Chevron Oil between 1974 and 1976^1^ defining the basin dimension and identifying potential petroleum structure and play. The stratigraphic column of the Muglad Basin have been divided into the Precambrian basement, the Upper Jurassic/Lower Cretaceous to Tertiary strata and the Tertiary to Quaternary sediments by (Fig. 2, Table 1)^1,10,11^. The lacustrine and/or floodplain shales of the lower Cretaceous non-marine Abu Gabra formation regarded as the major source rocks in the entire Muglad Basin (Figs. 2, 3, Table 1). The sandstones within the Bentiu, Aradeiba, Zarga and Ghazal formations represent the main reservoir intervals (Table 1). These reservoirs are sealed by overlying mudstone of the Darfur Group (Fig. 2). A chronicle of published literature in the Muglad Basin shows that extensive studies have been carried out on the organic richness of the source rocks in the basin, kerogen types have been determine as well, maturity, oil-source rock correlation and hydrocarbon generative potential of the Abu Gabra formation have been studied in details^4–9,12–15^.

**Table 1:**
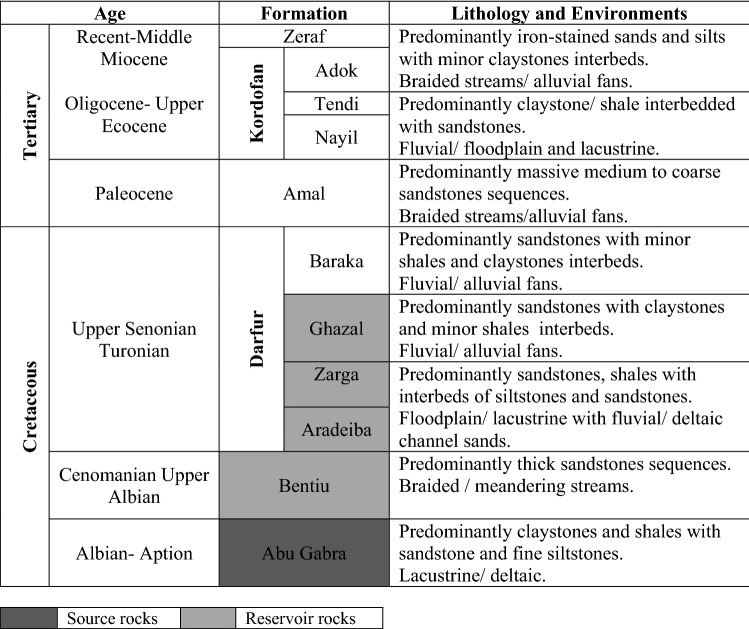
Stratigraphic units of the Muglad rift basin, SW Sudan, their lithology and depositional environment (adapted from Schull 1988).

The Zarga and Ghazal formations in the Muglad Basin have been identified as topmost reservoir horizons. These formations contain sandstone, siltstone, mudstone and shale lithologies, thus act as both reservoir and seal in the entire basin^1,3,16^. However, detailed investigation on the sedimentology, diagenesis and reservoir quality of the Zarga and Ghazal formations are yet to be carried out. This paper presents findings of an integrated sedimentary facies analysis, paleodepositional environment, petrographic analysis and petrophysical assessment of the two formations relating to their potential as petroleum reservoir.

### Geological setting

**Figure 4:**
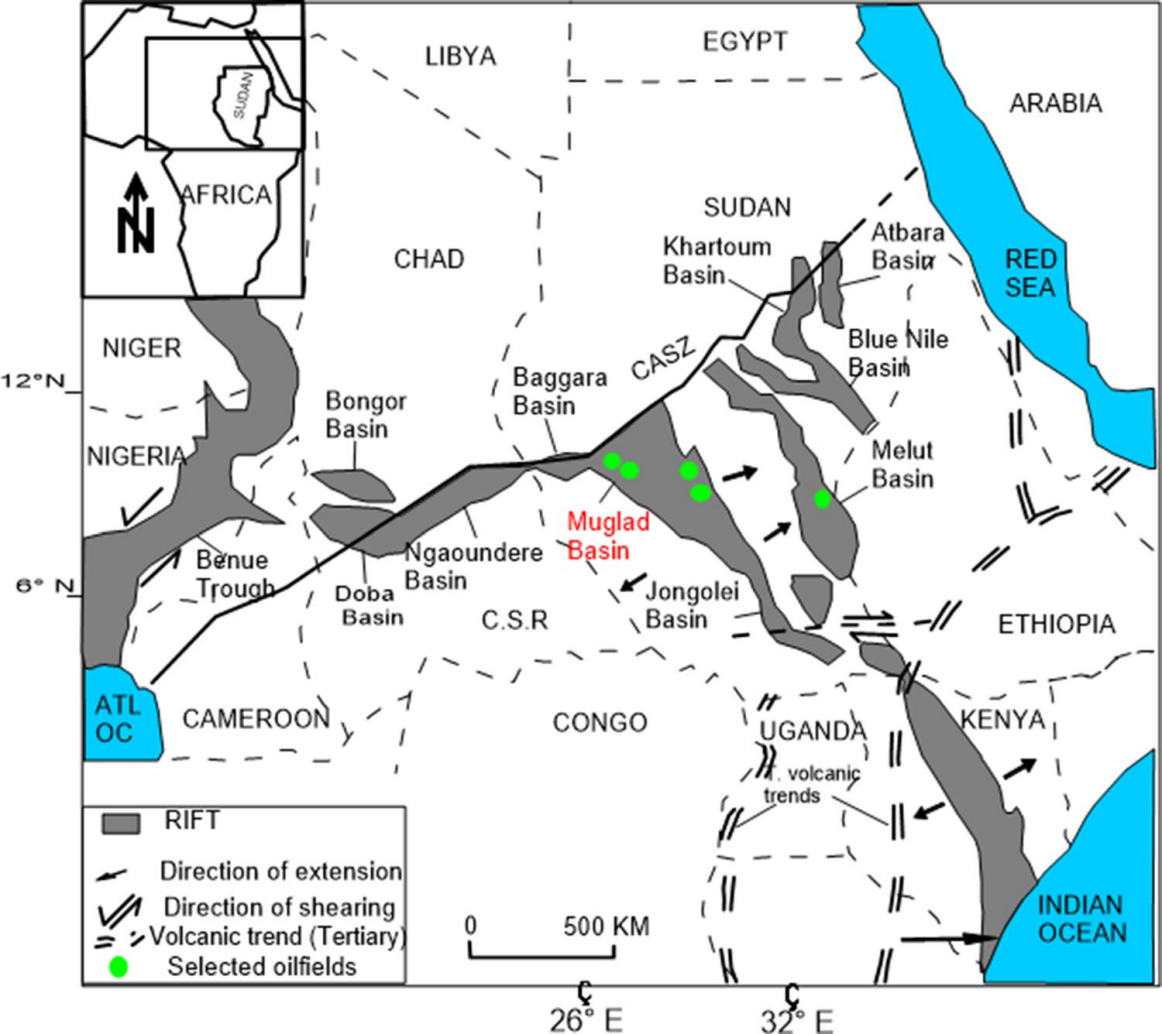
Location of the major basins across north north central Africa to the Benue Trough in Nigeria, through Chad and Sudan in relation to the Central African Shear Zone (created from information in reference^16^).

The evolution of the Central and Southern Sudanese basins began in the upper Jurassic to lower Cretaceous time was as a result of the tectonic processes that took place within the central and western continental margins of the African plate^1,7,8^. This is associated with the opening of the South Atlantic Ocean during the Cretaceous^7,14,17^. These processes occasioned the formation of the Central African Shear Zone (CASZ), which led to the development of the sedimentary basins in Sudan and its adjacent countries (Fig. 4). Based on regional geology, geophysical and well data, the structural development, of the Muglad Basin was divided into three rifting and sag phases dated 140 to 95 Ma, 95 to 65 Ma and 65 to 30 Ma (Fig. 2).

The initial rifting phase began in the upper Jurassic to lower Cretaceous and continue up to the Albian, together with several African rifts such as Benue, Anza, Ngaoundere and East Niger due to the initial opening of the South Atlantic Ocean (Fig. 4). The cessation of the rifting events in the Mugald Basin marked the deposition of thick sandstones of the Bentiu formation^1^. The occurrence of a dextral (right-lateral) reactivation along the CASZ in the upper Cretaceous resulted in the second rifting phase^17^. This rifting phase is characterized by widespread deposition of claystones interbedded with sandstones and siltstones which ended the deposition of the Bentiu formation. The termination of this rifting phase is recognized by thick sand sequence which ended with the sandstone of the Amal formation^1^. The third rifting phase occurred as a response to the initial opening of the Red Sea during the upper Eocene to Oligocene time^18^. This rifting phase is recognized by a thick sequence of floodplain and lacustrine sediments^18^. The sag phase represents a very gentle subsidence with little or no faulting and dominated by fluvial sediments^1^. During that period, the sedimentation in the Mugald Basin was mainly controlled by subsidence as a result of differential compaction of sediments^17^.

The major stratigraphic units in the Mugald Basin can be subdivided into the Precambrian basement, the upper Jurassic/Lower Cretaceous to Tertiary strata and the Tertiary to Quaternary sediments (Fig. 2). The basement rocks in the basin consists of granitic and granodioritic gneisses. These rocks have been dated to be ca. 550 Ma^1,3^. The upper Jurassic/lower Cretaceous to Tertiary non-marine strata/sediments are deposited in fluvial, alluvial fans, deltas, and lakes depositional environments. The non-marine and floodplain Abu Gabra formation was deposited during the Albian to Aptian. It consists of shales, claystones and sandstones (Figs. 2, 3, Table 1). The upper Albian to Cenomanian witnessed the deposition of the essentially thick sandstone sediments of the Bentiu formation from braided and meandering streams^1,3–5,7–9^.

The Turonian to lower Campanian represents a coarsening-upward sediments of the Darfur Group. In the Fula Sub-basin area (NE of the Mugald Basin), the Darfur Group is subdivided into Aradeiba, Zarga, Ghazal and Baraka formations^1,3–5,7–9^. The Aradeiba formation overlies the Bentiu formation and consists of mudstone, shale and thick sandstone beds (Fig. 2). The Zarga and Ghazal formations lie over the Aradeiba formation. These formations represent topmost reservoir horizon in the entire Muglad Basin and comprises dominantly of sandstone, siltstone, mudstone and shale lithologies deposited in fluvial and alluvial fan environments^1,3,16^. The Baraka formation comprises predominantly of thick sandstone interbedded with very thin beds of mudstone (Fig. 2, Table 1). The thick massive sandstone of Amal formation overlies, the Baraka formation, and it is in turn overlaid by the Kordofan Group, which consists mainly of lacustrine and fluvial claystones (Fig. 2, Table 1). This rifting ended the deposition of the Zeraf formation during the Lower Oligocene to Recent. The dominantly arenaceous Zeraf formation caps the stratigraphic column of the area and it was deposited in a braided fluvial setting. (Figs. 2, 3, Table 1).

### Methods of study

**Figure 5:**
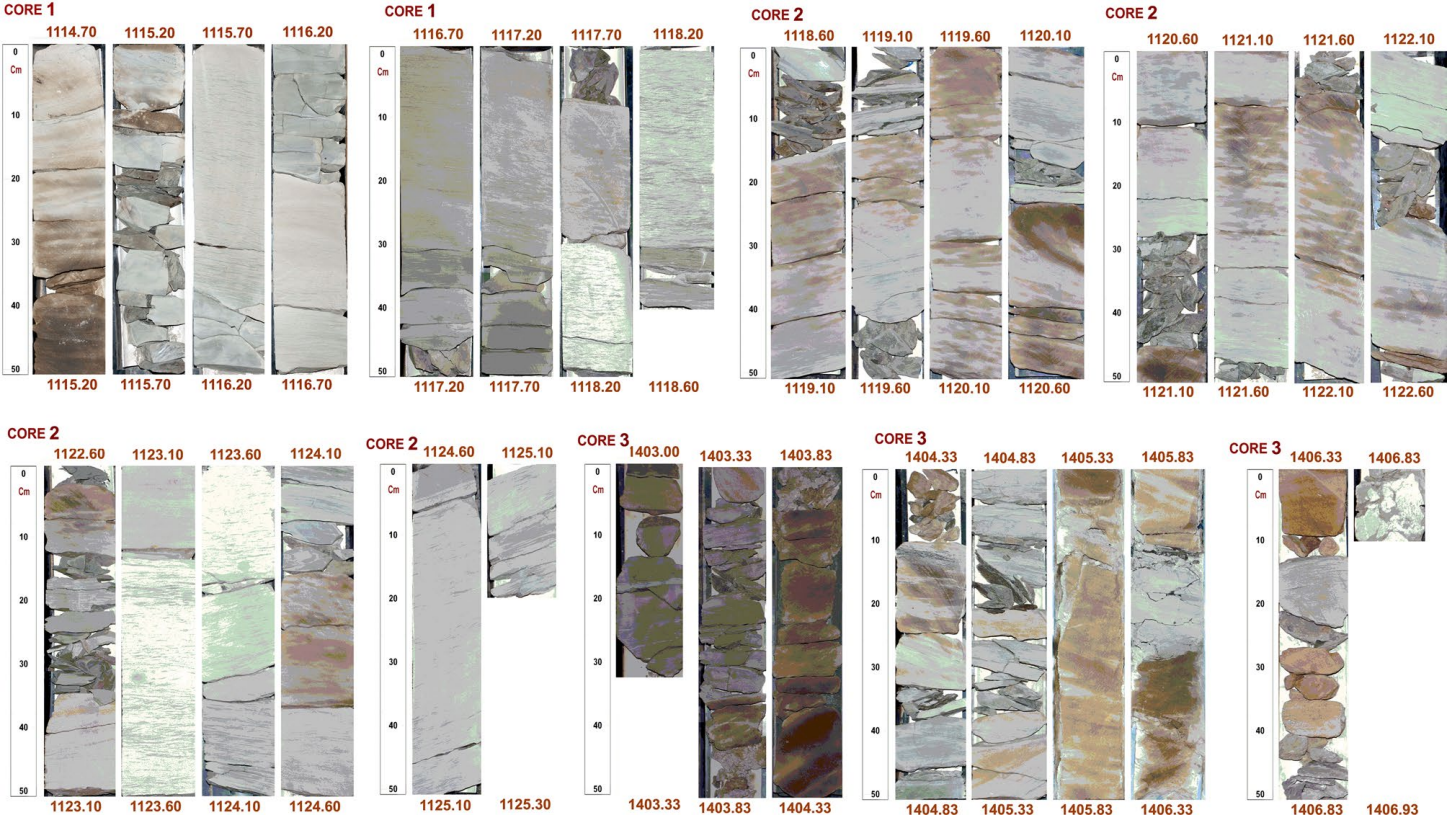
Conventional cores photos showing the studied core form Keyi N-A well.

**Figure 6:**
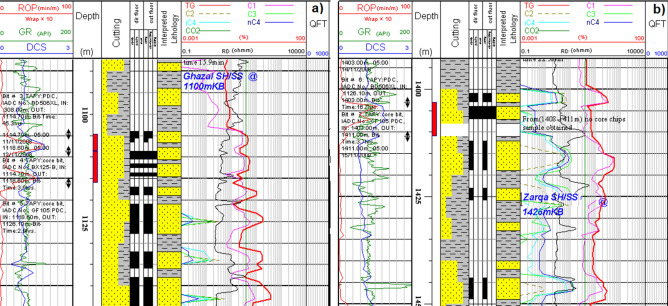
Well log response of Zarga (**a**) and Ghazal (**b**) formations sandstones.

An estimated 14.29 m subsurface cores representing three cored intervals (core-1, − 1114.70 to 1118.50 m, core-2, − 1118.50 to 1125.30 m and core-3, − 1403.30 to 1406.83 m) in the Keyi N-A well were used for this study (Fig. 5). The core-1 and 2 samples are from the Ghazal formation while core-3 samples are from the Zarga formation in the Muglad Basin. Apart from facies analysis, 14 core chips representing different lithofacies were subjected to further analysis as presented in Table 2. Lithological identification was based on physical observations and logging the cores (Fig. 5) against the gamma ray log at a vertical scale of 1:30 (Fig. 6). Facies analysis was defined based on sedimentary structures and lithological signatures observed in the cores. The thin section (TS) petrographic analysis was performed on the core chips (Table 2) using standard laboratory procedures. Vacuum impregnation with blue dyed resin was carried out to ease recognition of porosity. Carbonate minerals were identified by staining the samples with a mixture of Alizarin Red-S and potassium ferric cyanide solutions, while staining with sodium cobalt nitrate solution was done to aid identification of alkali feldspar. Thin section slides were point counted and individual percentages of the minerals were computed and presented on a data sheet. The classification of the rock type was based on^19,20^.

**Table 2:** Details of analysis carried out on the studied core samples.

Formation	Core no	Depth (m)	Facies type	Analyses					
				TS	SEM	GS	XRD		HM
							WR	CF	
Ghazal	Core-1	1114.85	St	2	1	2	1	1	1
		1116.70	Sr	2	1	1	1	1	1
	Core-2	1118.90	Sr	1	1	1	1	1	1
		1121.90	Sp	2	1	2	1	1	–
		1124.70	Sr	1	1	1	1	1	1
Zarga	Core-3	1404.10	Sm	2	1	1	1	1	1
		1405.50	St	2	1	2	1	1	1
		1406.50	Sm	2	1	2	1	1	1
Total				14	8	12	8	8	7

All depth references are core depths.

*TS* thin section analysis, *SEM* scanning electron microscopy, *GS* grain-size analysis, *XRD (WR)* X-ray diffraction analysis of the whole rock, *XRD (CF)* X-ray diffraction analysis of < 2 micron clay fraction, *HM* heavy mineral analysis.

**Figure 7:**
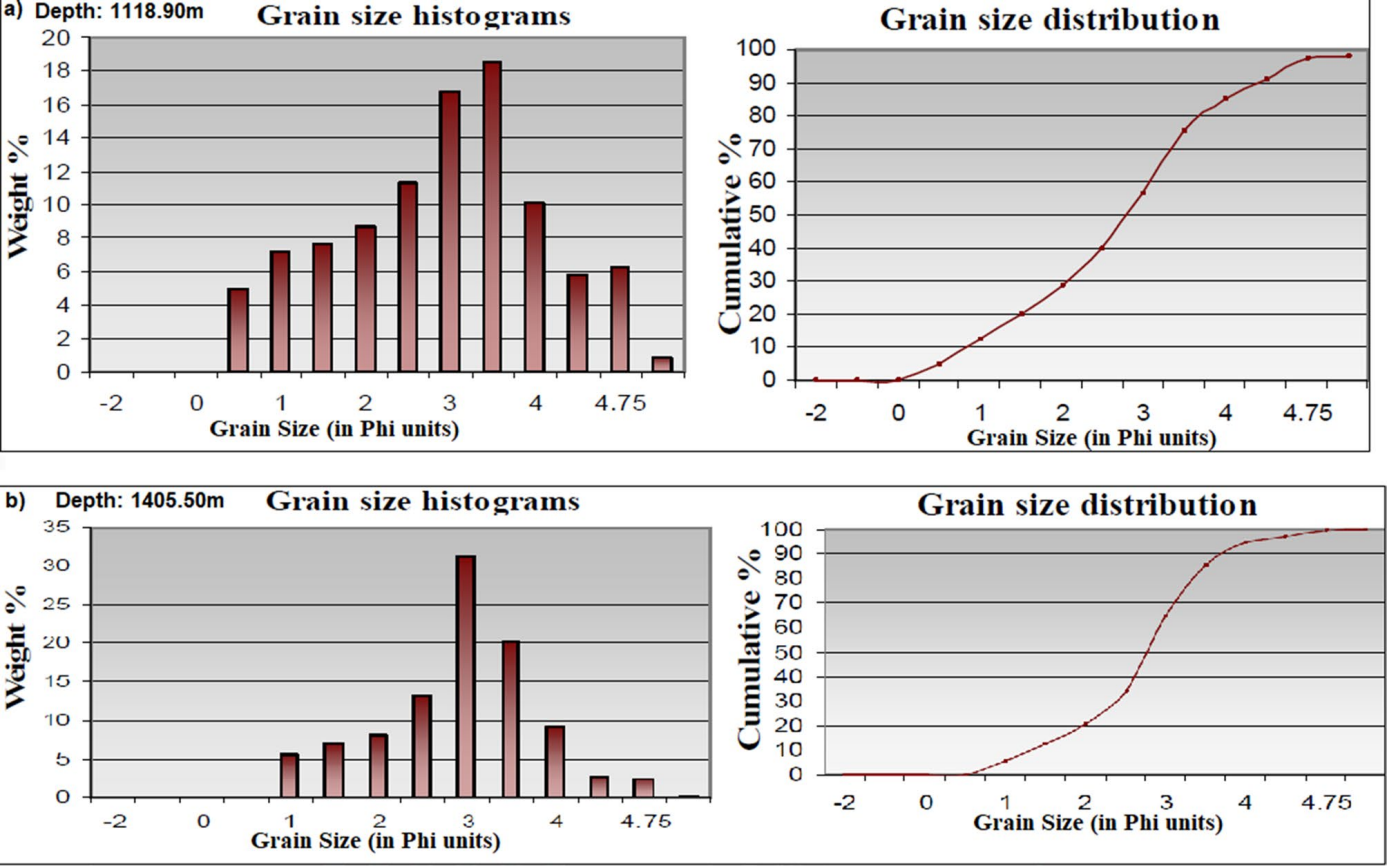
Grain size and sieve analyses histograms with cumulative weight percentage (curve in right) and grain size distribution (curve in left) for the studied Zarga (**a**) and Ghazal (**b**) formations.

Scanning electron microscopy (SEM) analysis was carried out on representative core samples to understand the pore geometry, composition, and morphology of the main pore-filling authigenic minerals in the reservoir. X-ray diffraction (XRD) analysis of the studied core intervals was based on previous workers’ standard^21,22^.**^19,20^. Also, both whole rock (WR) and the < 2 microns clay fraction (CF) component were carried out on eight cored chips (Table 1), while 12 representative samples (see Fig. 7) were used for grain size analysis. Heavy mineral separation was done with bromoform (2.89 g/cm^3^) following established procedures^23^ and the resultant slides were studied using polarizing microscope. The Jones and Roszelle method was adopted for permeability determination. Prior to this, the selected sample A (1118.90 m) was carefully chosen for the Flow-rate Dependency test to formation brine^24^. For permeability check, the flow rate was used to evaluate the base specific permeability so as to find out if there is any change in the permeability. Gas permeability was measured using calibrated steady state permeameter with nitrogen gas as the flowing medium. The sample was placed in a Hassler type core holder and the confining pressure used was 400 psi. A Klinkenberg correction was used for calibration of the permeability data obtained from the permeameter device.

## Results

### Facies description

**Figure 8:**
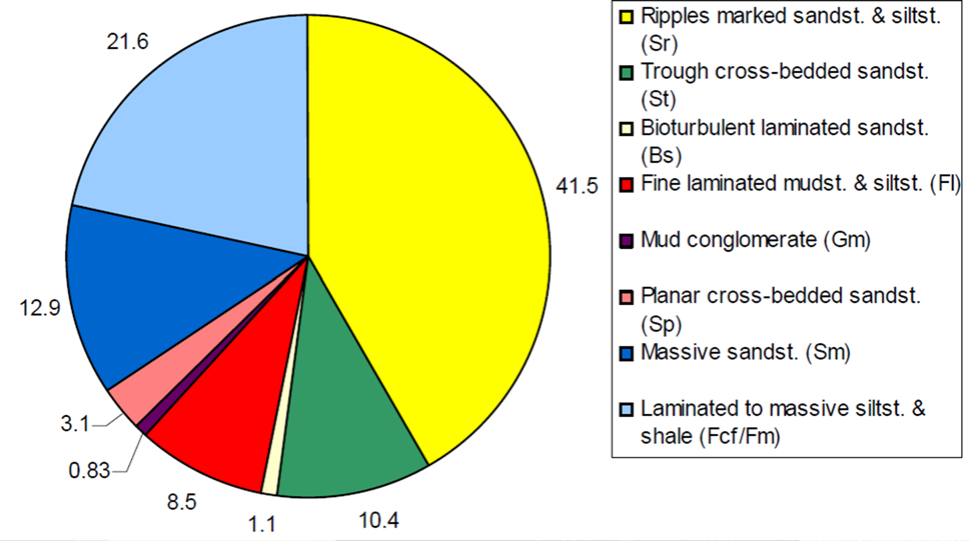
Pie chart of the different facies types in the studied core samples from Keyi N-A well.

In this study eight (8) different lithofacies were identified (Table 3) and represented on a pie chart as shown in Fig. 8. The facies types include.

**Table 3:** Facies parameters and inferred depositional environments of the cores from Keyi N-A well.

Facies code	Lithofacies	Facies characteristics	Facies description	Total thickness (m)	Percentage (%)	Interpretation of depositional environment
Sr	Ripples marked sandstone and siltstone	Light grey fine upper (FU) to fine lower (FL) ripple laminate facies	Coarse grains with low porosity and permeability	5.93	41.49	Ripples (top fluvial bar or deltaic mouth bar and deltaic distal bar)
St	Trough cross-bedded sandstone	Presence of cross stratification, grey grain of fine upper to medium lower grained (fU—mL) with red patches of few iron oxides, few mica and carbonate cements	Coarse grains with high porosity and permeability	1.49	10.42	Dunes (fluvial channel bar)
Bs	Bioturbated Laminated sandstone	Presence of bioturbations in the grey coloured fine upper grained (fU) with moderate porosity. It has some scattered mud clasts and oil shows	Coarse grains with high observed porosity and permeability	0.16	1.11	Deposits between deltaic distal bar and deltaic mouth bar
FI	Laminated mudstone and siltstone	Dark grey plane parallel to very low angle laminated fine grains associated with or without very small ripple marks	fine grains with low observed porosity and permeability	1.21	8.46	Overbank or waning flood deposits or delta distal bar deposits
Gm	Mud conglomerate	Grey Intraformational conglomeratic paticles with mud casts & crudely bebded gravel	Coarse grains with high observed porosity and permeability	0.12	0.83	Fluvial bars and/or natural levee deposits
Sp	Planar cross-bedded sandstone	Grey facies of fine upper grained with Planar(tabular) cross-bedding	Coarse grains with high porosity and permeability	0.45	3.14	Linguoids, transverse bars, sand waves (fluvial channel bars)
Sm	Massive sandstone	Structureless grey facies with fine upper (FU) to medium lower (mL) grains in a fining upward pattern It has oil show with some organic matter Porosity is reasonable high	Coarse grains with high porosity and permeability	1.85	12.94	Rapid sedimentation (high discharge event)
Fcf/Fm	Laminated to massive siltstone and shale	Fine grained dark grey matrix with partial lamination and partly massive showing some root casts The shale is fissile	fine grains with low observed porosity and permeability	3.08	21.55	Overbank (floodplain) deposits

#### Ripple marked sandstone and siltstone (SR)

The total thickness of this facies is 5.93 m as observed in core-1 and core-2 (Table 3, Fig. 5). It is light grey to grey colour sandstone with grain-size ranging from fine upper (FU) to fine lower (FL) while the grain roundness varies from sub rounded to well round. The grains are dominantly moderately sorted. Kaolinitic matrix is present and minor amount of carbonate cement has been identified. However, despite having the same characteristics with the sandstone, the rippled laminated siltstone beds have thickness of 0.60 m, which are lesser in thickness compare to the sandstone beds. Total porosity and permeability is generally low for the analyzed samples of Sr-facies, while the other facies (St Sp and Sm) are relatively high. This facies are similar to the “Sr” facies type in Miall’s classification. It is interpreted that the ripple marked sandstone beds were deposited on the top of fluvial bars while the rippled laminated siltstone beds were deposited on the deltaic distal bar^25^.

#### Laminated to massive mudstone and shale (FCF/FM)

This fine grained, kaolinitic rich facies is dark grey and well sorted with a total thickness of about 3.08 m present in the three studied cores samples. It is partially laminated and partially massive with some root casts which distinguish it from other facies such as Sr-facies. The shale component displays some degree of fissility. The facies has been described as “Fcf and Fm” according to Miall and interpreted as overbank (floodplain) deposits and deltaic distal bars^25^.

#### Massive sandstone (SM)

Massive sandstone facies type was observed in core-2 and core-3 with total thickness of 1.85 m (Table 3, Fig. 5). The facies is typically grey with grain-size between fine upper (FU) to medium lower (mL) having no evidence of lamination or ripple marks within a fining upward pattern. This well sorted grains range from rounded to sub-angular and often associated with mica and carbonate cements. An oil show with some organic matter has been observed as another feature of Sm-facies when compared to others (e.g. Fm and Sr-facies). Observed porosity is reasonable high for the sample analyzed. This massive sandstone resembles the “Sm” facies type in Miall’s classification and was formed by rapid sedimentation such as sheet-floods^25^.

#### Trough cross-bedded sandstone (ST)

This facies is present in core-1 and core-3 with a total thickness of 1.49 m (Table 3, Fig. 5). It is grey and the grain size varies from fine upper to medium lower grained (fU—mL). The grains are sub rounded to rounded with some sub-angular grains, and are well sorted. Few amounts of mica as well as carbonate cements and some few iron oxides were noticed in form of red patches. This typical trough cross-bedded facies shows relatively high total porosity and permeability in the studied cores. Thus, it could be classified as “St” type according to Miall and interpreted as the deposits of migrating 3-D dunes in a braided channel and deltaic mouth bars^25^.

#### Laminated mudstone and siltstone (FL)

Dark grey fine laminated facies (mudstone and siltstone) has a thickness of about 1.21 m found in core-2 and core-3 (Table 3, Fig. 5). A distinguishing feature of this facies is the presence of plane parallel to very low angle laminated mudstones and siltstones associated with or without very small ripple marks. This fine-grained laminated facies is classified as “FI” according to Miall^25^. It can be interpreted as overbank or waning flood deposits or delta distal bar deposits.

#### Planar cross-bedded sandstone (SP)

This facies is grey and present in core-2 with a total thickness of 0.45 m (Table 3, Fig. 5). The grains are fine upper grained (fU), sub-rounded, and well sorted. Some few carbonate and iron oxide cements were observed. Kaolinite represents the dominant matrix (clay). Observed porosity and permeability for the studied Sp-facies are also relatively higher (except the St-facies at 1114.85 m which compete with it). Planar (tabular) cross-bedding is a typical structure to recognize the sandstone. This facies is “SP” type according to Miall and interpreted to be linguoids, transverse bars or sand waves deposited in a fluvial channel^25^.

#### Bioturbated laminated sandstone (BS)

Bioturbated laminated sandstone facies is grey and present in core-1 with a total thickness of 0.16 m (Table 3, Fig. 5). The grain size is fine upper grained (fU). Analyzed grains are sub rounded to round and well sorted with observed moderate porosity. Aside reworking of by burrowing organism, a basic feature of this facies is the presence of some scattered mud clasts and oil shows in the studied samples. Thus, the facies is classified as “Bs” after Miall and interpreted as between a deltaic distal bar and deltaic mouth bar^25^.

#### Mud conglomerate (GM)

This facies is grey intraformational conglomerate and present only in core-3 with thickness of 0.12 m (Table 3, Fig. 5). A lot of mud casts were observed in this crudely bebded gravel. This facies could be classified as “Gm” type according to Miall and could be interpreted as the deposits of mouth bar^25^.

### Textural parameters

The grain-size analysis revealed that all the studied samples are dominantly of sand-size (< 0.09 mm to  > 1.00 mm) (e.g. Tables 4, 5, Fig. 7), with silt and clay components (< 0.09 mm) occurring in minor quantities. Generally, the < 250 μm (0.25 mm) and > 90 μm (0.09 mm) components dominate all studied samples. An estimated 83.33% of the analyzed samples are well sorted to moderately well sorted, while the rest are considerably poorly sorted (Table 6).

**Table 4:** Summary of grain size analysis.

Depth = 1118.90 m						Depth = 1510.55; Lithofacies = Sm					
Size grade	Sieve opening (mic)	Phi (Ǿ)	Weight (g)	Weight %	Cumulative %	Size grade	Sieve opening (mic)	Phi (Ǿ)	Weight (g)	Weight %	Cumulative %
Pebble	4000	− 2.00	0.00	0.00	0.00	Pebble	4000	− 2.00	0.00	0.00	0.00
Granule	2000	− 1.00	0.00	0.00	0.00	Granule	2000	− 1.00	0.00	0.00	0.00
V.C Sand	1000	0.00	0.00	0.00	0.00	V.C Sand	1000	0.00	0.03	0.08	0.08
C. Sand	710	0.50	2.48	6.20	6.20	C. Sand	710	0.50	2.00	5.00	5.8
	500	1.00	2.61	6.53	12.73		500	1.00	2.14	5.35	10.43
M. Sand	355	1.50	2.73	6.83	19.55	M. Sand	355	1.50	2.34	5.85	16.28
	250	2.00	3.25	8.13	27.68		250	2.00	3.83	9.58	25.85
F. Sand	180	2.50	5.47	13.68	41.35	F. Sand	180	2.50	17.96	44.90	70.75
	125	3.00	9.81	24.53	65.88		125	3.00	5.77	14.43	85.18
V.F. Sand	90	3.50	8.11	20.28	86.15	V.F. Sand	90	3.50	2.25	5.63	90.80
	63	4.00	2.85	7.13	93.28		63	4.00	1.74	4.35	95.15
Silt	45	4.50	0.83	2.08	95.35	Silt	45	4.50	0.55	1.38	96.53
	32	4.75	0.88	2.20	97.55		32	4.75	0.47	1.18	97.70
Pan	10 < 37	6.50	0.68	1.70	99.25	Pan	10 < 37	6.50	0.41	1.03	98.73
Sieve loss			0.30	0.75	100.00	Sieve loss			0.51	1.28	100
Total weight after sieve			39.70						39.49

**Table 5:** Statistical data of the analyzed samples grouped according to their facies types and association.

Facies group	No of samples	Minimum diameter (mm)	Maximum diameter (mm)
Rippled marked sandstone (Sr)	4	> 0.032	> 0.71
Trough cross-bedded (St)	3	> 0.032	> 1
Massive sandstone (Sm)	3	> 0.032	> 1
Planar cross-bedded (Sp)	2	> 0.032	> 0.5

**Table 6:** Petrographic data for the studied samples.

Depth (m)	Facies	Textural data					Rock name	Detrital mineralogy										Authigenic minerals						Porosity			Permeability (mD)
		Grain sorting	Grain contacts	Grain roundness	AV. pore connectivity	Pore types		Poly crystalline Qtz. %	Monocrystalline Qtz. %	Total Qtz %	Lithic fragment. %	K-feldspar %	Plagioclase %	Total feldspar %	Micas %	Heavy minerals %	Clay matrix %	Calcite cement %	Siderite cement %	Qtz over growth %	Pyrite cement %	Iron oxide cement %	Total cements	Primary %	Secondary %	Total porosity%	
1114.85	St	WS	CPLF	SR-SA	Good	PBP SWP SBP	Sub-feldspathic arenite	7.4	26.1	33.5	1.3	6.0	2.2	8.2	3.0	1.5	11.0	2.1	13.6	0.2	1.1	2.0	19.0	20.5	2.0	22.5	1196.71
1116.70	Sr	MS	FPC	SA-A	Fair-good		Sub-feldspathic arenite	2.6	21.5	26.1	2.0	4.2	2.4	6.6	7.6	0.6	28.0	2.0	5.2	0.1	2.2	2.4	10.9	17.1	2.1	19.2	161.87
1118.90	Sr	WS	CPLF	SR-SA	Good		Sub-feldspathic arenite	3.4	20.3	25.7	0.5	4.7	3.0	7.7	5.2	1.3	27.0	1.8	5.9	0.2	2.8	5.5	15.2	15.9	2.5	18.4	9.65
1121.90	Sp	MS-WS	PL	SA-SR	V.Good		Feldspathic arenite	5.1	25.2	33.3	2.7	6.0	2.3	8.3	3.5	0.9	13.9	2.0	3.6	0.5	1.7	7.3	15.1	20.4	2.9	23.3	1192.7
1123.50	Sp	MS-WS	PL	SA-SR	V.Good		Feldspathic arenite	9.4	23.7	34.1	1.2	7.1	2.0	10.1	5.4	0.6	13.2	2.6	6.2	0.6	2.7	0.9	17.0	20.4	3.0	23.4	1102.45
1124.00	St	WS	CPLF	SR-SA	Good		Feldspathic arenite	7.8	23.2	31.0	1.2	5.3	2.2	7.5	4.7	0.8	12.5	2.4	11.0	0.8	1.1	2.7	17.8	20.3	3.9	24.5	1100.5
1124.70	Sr	PS	PL	SA-SR	Good		Feldspathic arenite	5.0	19.3	26.3	1.0	5.0	2.2	7.2	2.2	2.0	27.0	2.8	4.0	0.7	1.6	7.3	16.4	15.5	2.4	17.9	48.5
1404.10	Sm	MS-WS	PL	SA-SR	V.Good		Feldspathic arenite	13.8	25.0	38.8	1.1	6.1	3.2	9.3	1.4	1.4	9.0	2.0	2.4	1.0	0.5	2.4	8.3	26.0	4.7	30.7	1059.2
1404.30	Sm	MS-WS	PLS	SA-SR	Excellent		Sub-feldspathic arenite	13	25.0	38.0	0.8	6.2	2.0	8.2	2.3	0.9	9.2	1.6	4.1	0.9	1.1	2.4	10.1	25.6	4.9	30.5	1090.45
1405.50	St	MS-WS	PCLS	SR-R	Good		Sub-feldspathic arenite	11.1	22.1	35.2	2.0	5.2	4.4	9.6	3.0	1.7	8.4	1.7	4.1	1.5	1.5	1.9	10.2	24.4	5.0	29.4	1057.53
1406.00	Sm	MS-WS	FPL	SR-SA	V.good		Sub-feldspathic arenite	12.0	22.1	34.1	0.7	7.2	2.8	9.0	3.2	2.0	8.8	1.1	4.5	2.0	0.6	4.0	12.2	25.0	5.0	30.0	1051.09
1406.50	Sm	MS-WS	FPL	SR-SA	V.good		Feldspathic arenite	11.2	26.2	36.4	1.3	7.3	3.0	10.3	2.9	1.0	8.9	1.4	5.4	1.9	0.6	2.2	11.5	22.2	5.5	27.7	1070.45

*WS* well sorted, *MS* moderately sorted, *PS* poorly sorted, *C* concavo-convex, *P* point, *L* long, *F* floating grains, *S* sutured, *PBP* primary interparticle, *SWP* secondary intraparticle, *SBP* secondary interparticle.

### Petrography

The source rock provenance determines the sandstone composition. Petrography, the sandstone contains quartz, feldspar, micas (mainly biotite and muscovite). Also present are rock fragment, opaque and heavy minerals as well as authigenic components such as iron oxides, quartz overgrowth, siderite cement, and authigenic clays (Table 6). These minerals and the authigenic components are highlighted below.

#### Quartz

**Figure 9:**
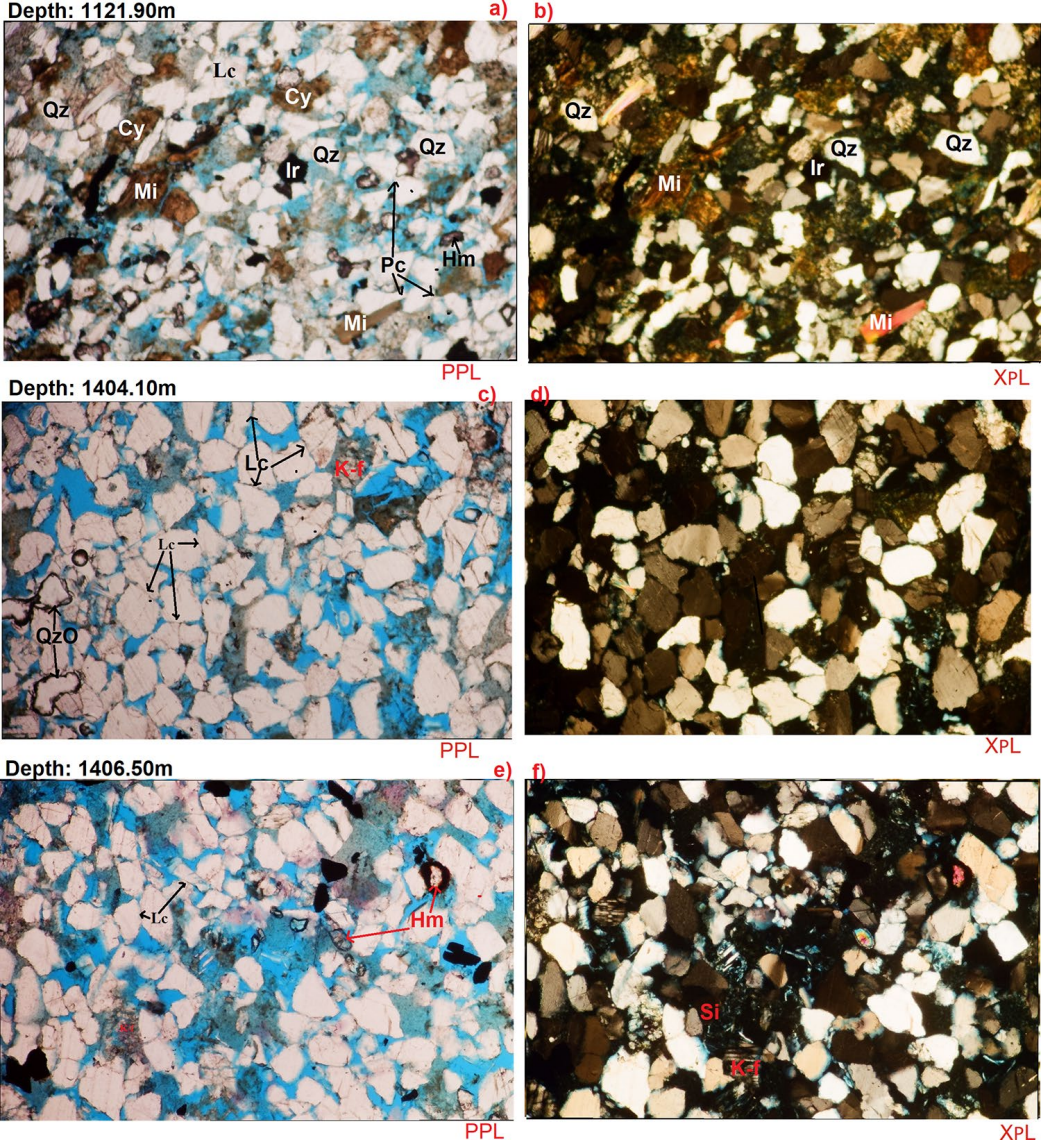
(**a**–**f**) Fine to grained, well sorted, sub rounded to sub angular, patchy cemented, and moderately compacted with point, concavo-convex and long grain contacts Sub Feldspathic Arenite, common monocrystalline and polycrystalline quartz (**a**–**f**). Some quantities of mica (**a**,**b**) which arranged along the bedding plane due to the hydraulic fraction of the mica flakes as well as very few heavy minerals (**a**,**e**). Considerable amount of detrital clays occupying some pore spaces (**a**,**b**). Some quantities of iron oxides cement occur as pore filling (**a**,**b**), minor amount of quartz overgrowths (**c**) as well as with some amount of siderite (**f**). *Qz* quartz, *QzO* quartz overgrowth, *Lc* long grain contact, *Pc* point contact, *Cy* clay, *Mi* mica, *Ir* iron oxide, *Hm* heavy mineral, *K-f* K-feldspars, *Si* siderite.

It is the most common mineral in the sandstones appearing as dull grains having weak birefringence and low refractive index that is only slightly higher than that of the mounting medium^26,27^. Monocrystalline quartz (Qm) and polycrystalline quartz (Qp) were seen. Monocrystalline quartz (20.3–26.2%) is more in abundance than the polycrystalline quartz (2.6% to 13.8%) as shown in Table 6. Fractured quartz grains are also common. The quartz grains are mainly sub rounded to sub angular and are well sorted to moderately sorted (Table 6, Fig. 9a–f). Most of the quartz crystals exhibit undulose extinction with some of the polycrystalline quartz grains being granular and having sutured boundaries. The quartz grains coalesce minerals such as rutile, zircon, iron oxides and tourmaline (Fig. 9).

#### Feldspars

K-feldspars (Fk) such as orthoclase (Or), microcline (Mi) and perthite (Pe) are the dominant feldspars observed with relative abundances of 4.2% to 7.3%, while Plagioclase feldspar range between 2.2 and 4.4% (Table 6). The K-feldspar shows different degrees of alteration and dissolution ranging from relatively fresh, unaltered grains to partly to almost completely dissolve (Fig. 9c–f). Predomination of K-feldspar over plagioclase feldspar could have resulted from the preferential breakdown of the latter as the former characteristically have more stable chemical stability^28^. In addition, K-feldspar is much more common in continental basement rocks (acid gneisses)^28^, which are probably the provenance of many sandstones in the study area.

#### Micas

Biotite (Bi) and muscovite (Mu) were identified by their platy cleavage and parallel extinction. Biotite has a brown to green pleochroism while muscovite appears dull under plane-polarized light, but with bright second-order color under crossed polarized light^26^. The concentration of the mica varies in the examined samples, the highest was at 1116.7 m having 7.6% (Table 6, Fig. 9a,b). These micas generally occur as large detrital flakes concentrated along partings, laminae, and bedding planes. The distribution is primarily a function of sorting and is determined by the hydraulic behavior of the mica flakes^28^. Also, few micas shows curvilinear structure indicating moderate compaction.

#### Opaques

These accessory detrital components occur in minor amounts (trace quantities). Opaque are mainly hematite and pyrite. Opaque grains are very fine to medium grained and show moderate abrasion.

#### Heavy minerals

Microscopic investigation of 7 samples from Zarga and Ghazal formations in the studied intervals, a few major heavy minerals were identified. They occur in minor amount in all the studied samples. Heavy Mineral concentration ranges between 0.6 and 2.0%. Moreover, they have been observed as free grains and/or enclosed inside the quartz grains (e.g. Fig. 9e).

#### Detrital clays

Detrital clays have been recorded in all the studied samples, where their relative abundances vary between 8.4 and 23.0% that recorded at depths of 1405.50 m and 1124.70 m, respectively. However, thin section analysis suggests that the vast majority of the interstitial clays are authigenic in origin particularly the kaolinite.

#### Carbonates

Siderite (Sid) and calcite (Ca) were noticed as patchy cements in all the observed samples (Table 6). Calcite and siderite abundance are up to 2.8% at 1124.70 m and 13.6% at 1114.85 m. Both minerals can be identified by their perfect rhombohedral cleavage and a pearl grey or white high order interference color. Calcite is dull, but often cloudy, usually anhedral whereas, siderite resembles calcite but may often be distinguished by brown stains around the borders of the grains and along cleavage zone^26^.

#### Quartz overgrowths

Well developed syntaxial quartz overgrowths are recorded in almost all the analyzed samples ranging between minor amount and 2% (Table 6, Fig. 9c). Thin section observations revealed the presence of well-developed euhedral, quartz overgrowths.

#### Iron oxides cement

Iron oxides are widely spread in the examined samples and they were recorded as a cementing material. However, the higher percentage which is 7.3% was recorded at depths of 1121.90 m and 1124.70 m (Table 6).

#### Pyrite

Authigenic pyrite as cementing and replace agent has been noticed in all the studied samples (Table 6). Through the core description, pyrite nodules were recorded as cement filling within the pores. SEM analysis shows presences of patchy aggregates of sub cubic to cubic pyrite as micro crystals.

#### Scanning electron microscopy (SEM)

**Figure 10:**
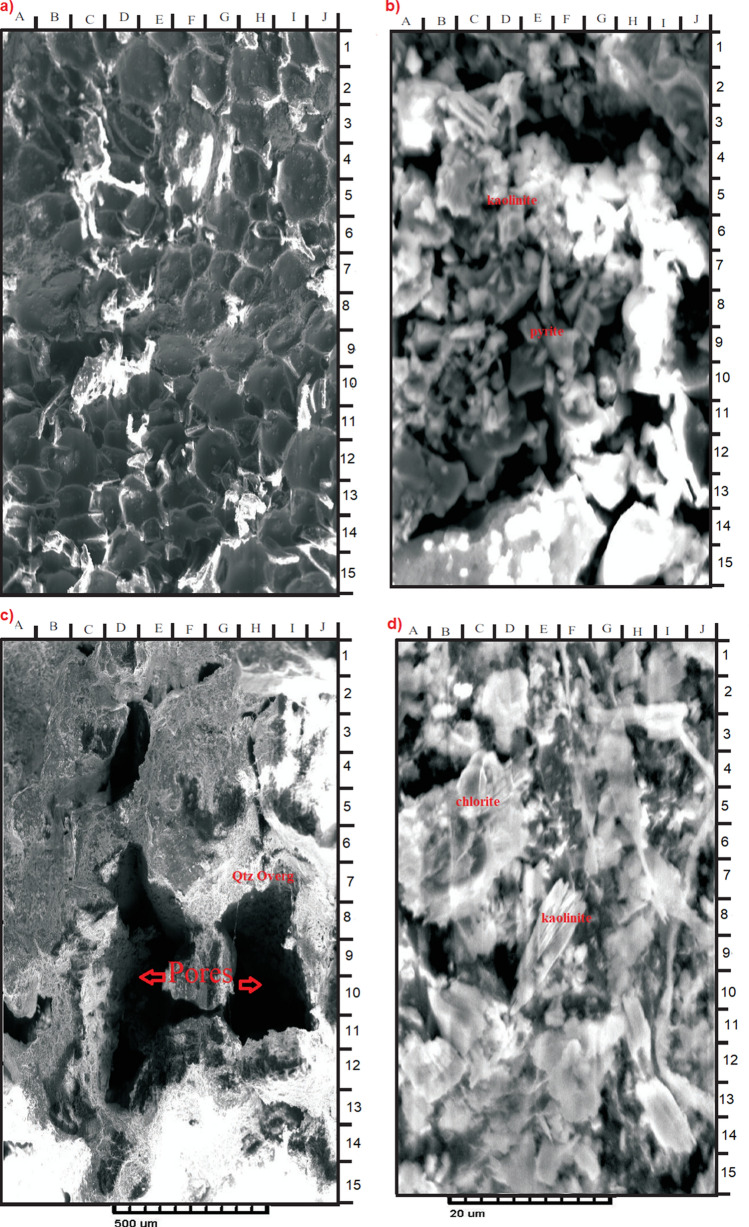
(**a**) High cemented with relative closed grain packing and low degree of intergranular porosity. (**b**) Few amounts of stacked pseudo hexagonal plates of kaolinite partially filling a pore ((**b**) E–F 5–6). Considerable amount of pyrite, which observed within the pores ((**b**) C–F 7–8; E–F 11–12). Few quantities of iron oxides occur between the detrital grains ((**b**) B–C 6; F7). Minor amount of carbonate ((**b**) F–G 8–9). (**c**) Moderately cemented with relatively open grain packing and medium degree of primary intergranular porosity ((**c**) D–E 2–5 & D–G 6–12). (**d**) Common corroded detrital plates of kaolinite partially filling some pores and partially cover some detrital grains ((**d**) C–D 2–4 & E–H 11–13). Some authigenic kaolinite appears as pseudohexogonal plates or books arranged faceto- face into an elongated stacked form called verm (**d**): D–G 7–10 & A–B 11–12) Some rose-like shape of chlorite ((**d**) A–D 3–7).

The SEM analysis involved a detailed investigation and description for the sample material with a special focus on the pore geometry, composition as well as on the morphology of the main pore-filling authigenic minerals. Kaolinite, pyrite, iron oxides and carbonate minerals (Fig. 10) are identified in the samples. The characteristics of the studied samples are further illustrated by two photomicrographs for each examined sample (e.g. Figure 10) in order to clarify the diagenetic effects of the clay minerals on the reservoir quality.

### Clay minerals

Eight clay rich samples from the studied intervals have been analyzed and five clay mineral species were identified from the size fraction less than 2 microns. The five clay minerals species are: Kaolinite, Chlorite, illite, smectite and smectite/illite (Table 7).

**Table 7:** Percentages of the clay minerals in the analyzed samples.

Core no	Sample depth (m)	Clay minerals %				
		Kaolinite	Smectite	Illite	Chlorite	Illite/chlorite
1	1114.85	41.5	0.14	0.93	19.0	0.07
	1116.70	59.9	2.04	3.79	34.1	0.15
2	1118.90	75.2	0.83	1.34	22.5	0.10
	1121.90	86.4	0.09	0.41	13.1	0.09
	1124.70	49.1	0.10	0.44	50.3	0.10
3	1404.10	85.0	0.06	0.25	14.6	0.06
	1405.50	79.8	0.14	0.93	19.0	0.07
	1406.50	82.5	0.05	2.43	14.9	0.10

#### Kaolinite

This is common in all the examined sandstones in varying concentration between 41.5 and 86.4% (Table 7). Kaolinite forms mostly in surficial environment through pedogenetic processes^29^. It may also occur in water environment from the alteration of K-feldspar in acid organic rich waters^28^. Moreover, hydrothermal alteration of aluminosilicates, especially of feldspars may also form kaolinite^19,20^. According to Keller and Weaver, formation of detrital kaolin minerals required presence of H+ ions and efficient removal of metal cations^30,31^. These conditions are favored by strong leaching in the source area, which implies abundant rainfall, permeable rocks and favorable topography, and hence, evacuation of the Ca, Mg, Na and K ions. Climatic conditions favorable for the formation of kaolin mineral are essentially tropical and subtropical.

**Figure 11:**
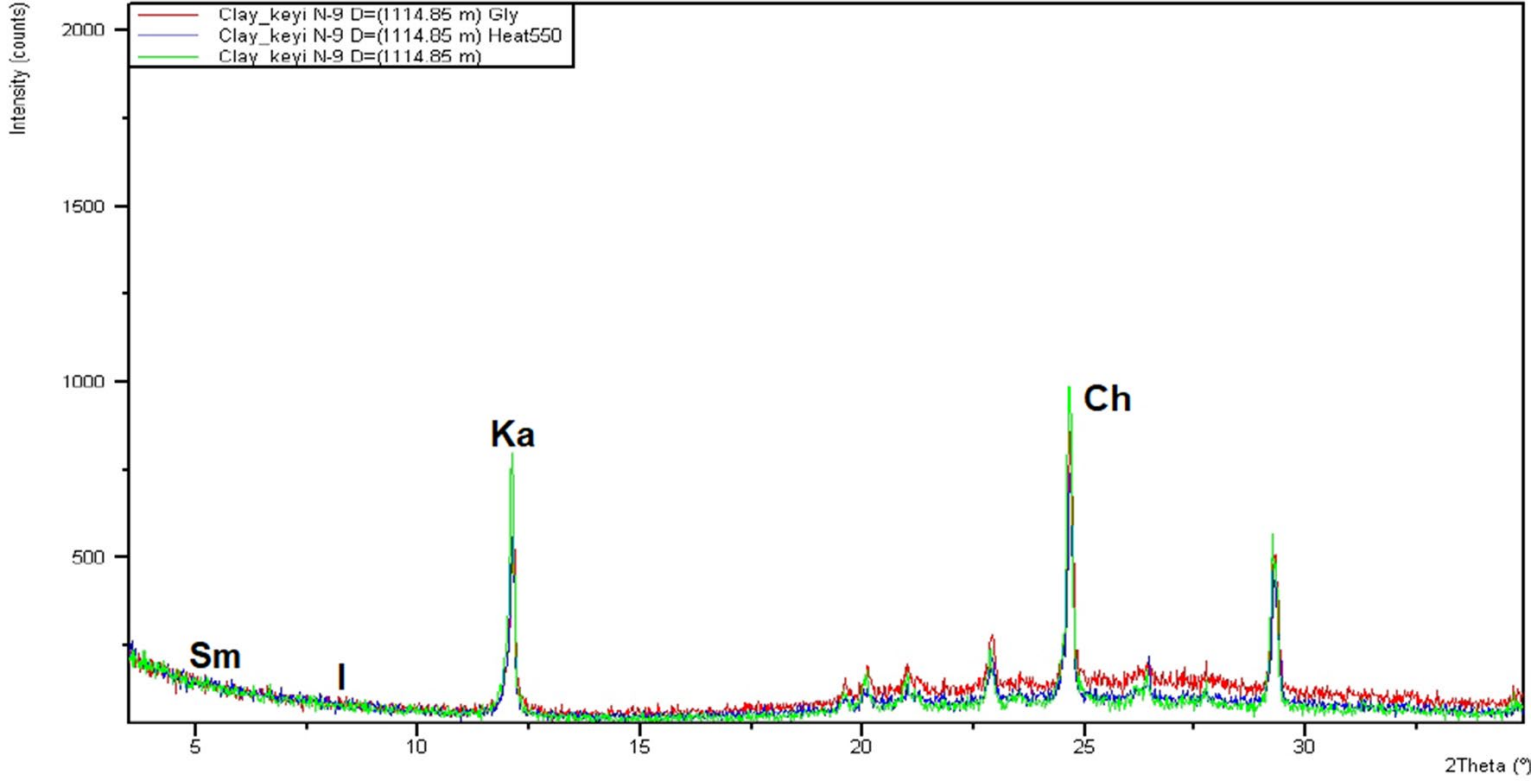
Representative XRD graphical charts showing the degree of crystallinity of the clay minerals present in the studied core samples.

The sharp peaks of the kaolinite in most of the XRD charts (e.g. Fig. 11) implies great part of the kaolinite is monocrystalline^31^, have been authigenically formed. However, other part of the kaolinite in the studied intervals is detrital formed by hydrolytic processes and have been confirmed by the relatively flattened kaolinite peaks in the XRD (Fig. 11).

### Chlorite

Chlorite concentration ranges between 13.1 and 56.5% (Table 7). Petrographic investigation shows chloritization (Fig. 10d), where chlorite clay mineral fills up pore spaces between grains. Chlorites are considered either as a 2:1 or as a 2:1:1-layer group with a hydroxide interlayer. Their typical structure shows a regular alternation of negatively charged trioctahedral micaceous layers and positively charged octahedral film. Part of the chlorite within the examined intervals is detrital and derived from biotite-rich metamorphic source rocks during the intermittent hot dry periods. However, other part is authigenic formed by the alteration of the biotite as revealed by the petrographic analysis of sample depth 1121.90 m (Fig. 9a).

### Smectite

Clay mineral abundance is between 0.05 and 2% (Table 7). According to Weaver, the climatic and topographic determinants essential for the formation of smectite are basically the opposite to those which favors the formation of kaolinite^31^. Smectite is in most cases formed in low relief areas where poor drainage prevents the silica and the alkaline earth ions such as K^+^, Na^+^, Ca^+2^ and Mg^+2^ from being leached out. Moreover, smectite is formed from the weathering of basic and ultra-basic rocks or their metamorphic equivalents in areas of low rainfall, low water flux and low temperature. The smectite peaks are not sharp as in kaolinites, as such this implies greater part of the smectite is polycrystalline reflecting detrital occurrence (Fig. 11). This detrital smectite was formed during the intermittent hot dry season prevailing at the source area. However, the prevailing low content of the smectite in these cored intervals could be due to the late transformation of the smectite to illite, as well as to chlorite.

## Discussion

### Sedimentological and petrographical characteristics

The core samples from the Keyi N-A Well comprises essentially of continental clastics. The succession composes dominantly of sandstone facies (Sr, Sm, St, Sp & Bs facies). The studied core-3 and core-1 intervals display coarsening upward sequences, whereas core-2 displays a dominantly fining upward sequence (Fig. 5). The fining upward succession starts with a fine-grained facies (ripple laminated sandstone beds), and then passes dominantly to massive mudstone and shale beds. However, in few cases the fining upward sequences start with fine grained massive, planar, or trough cross-bedded sandstone facies, and typically end with mudstone beds (Fig. 5). The thickness of the fining upward sequences range between 0.20 and 1.16 m (Fig. 5 core-2). The coarsening upward sequences start mainly with massive to laminated mudstone beds, and end with fine to medium grain-size ripple marked or trough cross-bedded sandstone facies (Fig. 5). The thickness of the coarsening upward successions varies between 0.35 and 1.52 m (Fig. 5 core-1 and 3).

The coarsening upward sedimentary sequences in Zarga formation typically start with a massive mudstone or laminated mudstone interval, and end with massive medium sandstone or trough cross-bedded fine-grained sandstone (Fig. 5). The sedimentary succession in core-1 and 2 of the Ghazal formation suggests deposition in a fluvial system that was later predominantly succeeded by deltaic depositional environment, whereas the sedimentary signatures in the Zarga formation (core-3) indicate a dominant deltaic depositional setting.

Sedimentological characteristics of the studied cores led to the identification of eight facies assemblages: the ripple mark sandstone and siltstone Sr, massive mudstone and shale Fm, massive sandstone Sm, trough cross-bedded sandstone St, laminated siltstone and mudstone FI, planar stratified sandstone SP, bioturbated sandstone Bs, and mud conglomerates Gm (Table 3).

**Figure 12:**
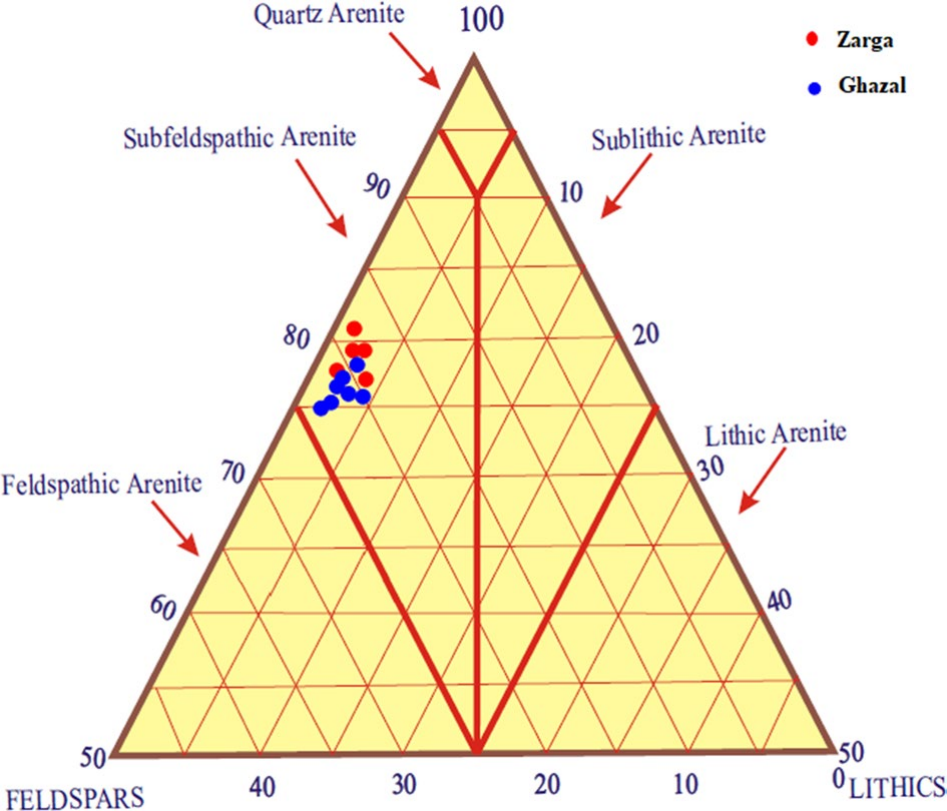
QFL Ternary Diagram for Sandstone Classification of the Zarga and Ghazal Formations sandstones (created from information in reference^33^). *QFL* Quartz–Feldspars–Lithics.

**Figure 13:**
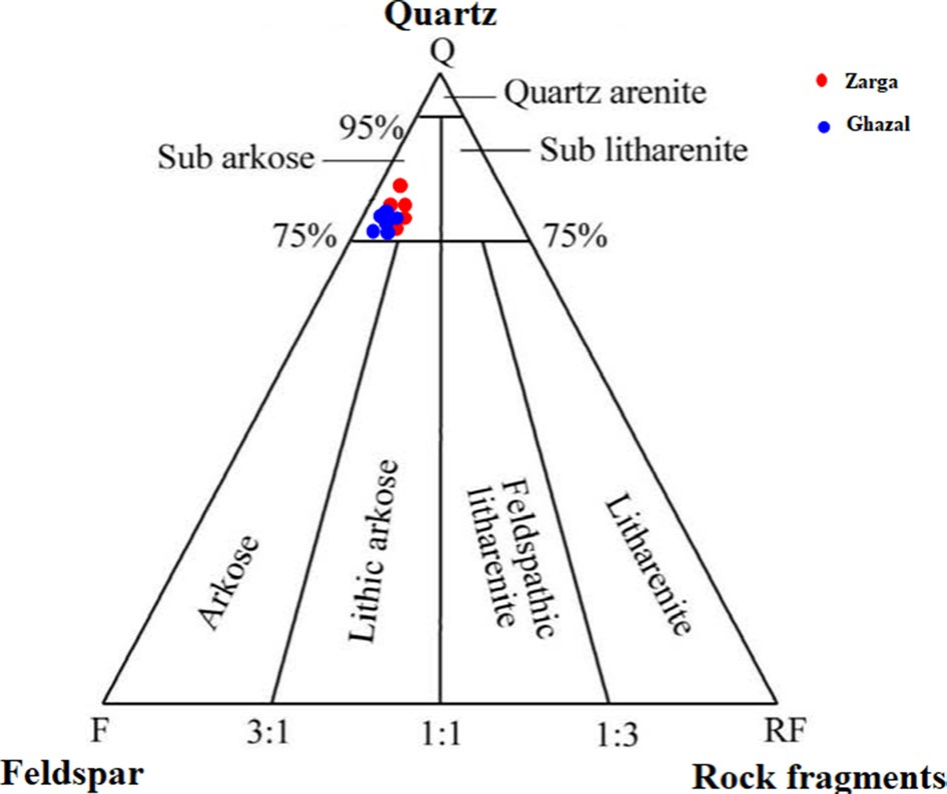
Classification of sandstones in the study area (created from information in reference^33^).

The analyzed samples are somewhat rich in quartz and feldspars, with less lithics or rock fragments. Using the Dott^32^ and Pettijohn et al.^33^ classification schemes, the studied sandstone facies plotted under the sub-feldspathic arenite (Fig. 12) and subarkose fields (Fig. 13), respectively. The preponderance of detrital and authigenic components in the sandstone are in the order of (from abundant to least abundant) mono-crystalline quartz polycrystalline quartz, feldspar, mica, detrital clay, authigenic clay, and carbonaceous remains. Other accessory materials like lithic fragment, iron oxide and heavy mineral occur in trace amounts within the analyzed samples (Table 3).

**Figure 14:**
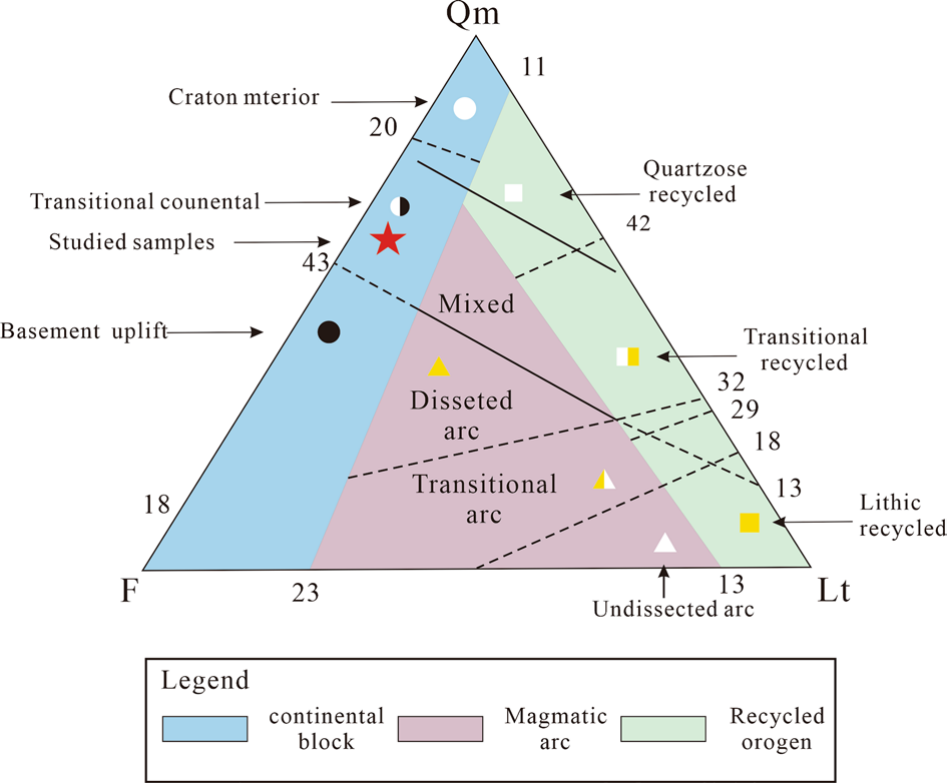
Tectonic setting of the studied sandstone (created from information in reference^37^).

Kaolinite is present as authigenic constituent, while varying quantity of carbonates occurs with insignificant amount of quartz overgrowths in majority of the analyzed cores (Table 6, Fig. 10). The Sandstone grains are sub-rounded to sub-angular, fairly compacted, and exhibit concavo-convex, long and float grain-to-grain contacts (Fig. 9). On the other hand, sutured grains, which portray much compactional influence were noticed in minor samples. Pore types in the analyzed samples are dominantly primary and secondary interparticle forms^8^. The secondary intraparticle porosity was derived after partial dissolution of the detrital feldspars. The pores are often filled with kaolinite, carbonates, and quartz; thereby reducing the porosity (Fig. 10). In the Zarga formation (Core-1 and 2) pore point counts range from 17.9 to 24.5% (e.g. facies Sr & Sp,), whereas pore counts in the Ghazal formation sample (core-3) vary between 27.7 and 30.7%, with average pore sizes that are highly variable ranging from 25 to 345 microns (e.g. facies Sm). The pore interconnectivity ranges from fair to good in the Ghazal formation, while the Zarga formation has pore interconnectivity that is good to very good. Thus, better reservoir quality is implied for the Ghazal formation reservoir sandstones in contrast to the Zarga formation reservoirs. Modal analyses of the sandstone depict its origin from continental provenance, transitional within stable interior of a craton and basement uplift that is a basement area of generally elevated along rifts (Fig. 14).

Clay mineral analysis highlights kaolinite, chlorite, illite, smectite and smectite/ illite as the main clay mineral constituents of the studied samples (Table 5, Fig. 10). XRD charts shows actuate peak kaolinitic monocrystalline signifying it was authigenically formed, although few of the kaolinites are detrital. Chlorite content is partly authigenic and partly detrital in origin. Lower horizons have higher amounts of kaolinite implying leaching and chemical weathering effects in humid warm climatic condition, whereas the upper horizon contains high chlorite and smectites due to hot dry condition.

Grain-size distribution analysis of the sandstone samples indicates that all examined samples contains sand-size fractions (> 0.063–0.5 mm), while silt and clay size (< 0.063 mm) particles occur in lesser amount across all facies except for the Sr facies, which shows significant clay content up to 28%. Exceptionally, three samples contain components with grain-sizes greater than 1.00 mm. Moreover, components of grain-sizes range between more than 250 μm and greater than 63 μm (> 0.063 to > 0.250 mm) exemplifying the dominant constituents in all the samples. Similarly, granulometric analysis shows that four samples are well-sorted, one sample is poorly sorted while the rest samples are moderately to well-sorted.

### Grain textural features

Grain textural features provide information on the geometry of individual grains including grain sizes, grain shapes, sorting, and packing. These geometrical attributes have direct effect on primary porosity and permeability, even though porosity is independent of grain sizes, whereas permeability decreases with decreasing grain sizes^8^. Chen et al. pointed out that coarse grain sizes give rise to higher porosity values than the fine grained particles^34^. Most of the examined Zarga formation samples are characterize by coarse grain with occasional medium grain sizes. The total clay content in this formation ranges from 8.4 to 9.2% whereas Ghazal formation clay content is from 11.0 to 28.0% (Table 6). Facies Sr has finer grain sizes than the other facies having low clay content. Porosity increases with degree of roundedness, and rounded grains are more porous than angular particles. Greater porosity values are recorded for highly anisometric particles^8,26^. The grain roundness in all the studied samples is less variable (Fig. 9a–f). Most of the samples are sub-rounded to sub-angular in shape, while only few samples show varying degree of roundness (Table 6). Isometric particles are observed in the samples dominated by very fine grain particles and high total clay content (facies Sr). Similarly, increasing sorting result in higher porosity and permeability. In poorly sorted sediments, clayey and very fine grain sizes could be found between larger grains thereby decreasing the porosity and permeability^8,35,36^. The sorting pattern of the sandstones is between moderately to well-sorted with few samples being poorly sorted. Porosity and permeability decrease further by deformation and packing of the sediment grains. Loosely cemented particles have high porosity and less grain-to-grain contacts^8,36^. Majority of the studied sandstones are less compacted as shown in Fig. 5a–f. Also, grain-to-grain contact are displayed significantly well in the less clay rich samples.

### Provenance of the sediment

The percentage of the different sandstone grains are plotted on a triangular diagram for modal analysis, which depicts their provenances (Fig. 14). The modified Q_m_FL triangular plot of Dickinson et al., shows the monocrystalline quartz grain, the feldspars and the lithic remains which emphasis maturity of the sediment^37^. This diagram plays a role in classifying sandstones from different tectonic settings (Fig. 14). The triangular plot illustrates that the sandstones originate through transitional provenance around the continental part within the stable interior of the craton and the basement uplift along rifts (Fig. 14). This enables the detrital constituents to be reclaimed after being moved for quite a long distance and accumulated in an extensional and pull-apart basin. Paucity of siderites, calcites and iron oxides besides their co-existence suggest that the lake system was hydrologically open and maximum lake level was imposed by an outlet having relatively stable water level seaside.

### Diagenetic processes

Petrographic analyses of samples from the Zarga and Ghazal formations indicated that diagenetic signatures affected their syndepositional porosity and permeability thereby altering reservoir quality. Identified factors that increase porosity and permeability are dissolution of feldspar and mica, partial dissolution of carbonate (calcite) cement and clay. Contrastingly, the porosity and permeability reducing processes are the presence of detrital clays, kaolinization, iron oxide precipitation, carbonate cementation, compaction, quartz overgrowths and pyrite cementation^8^.

The richness of detrital clay minerals, which reduces the porosity and permeability within the Zarga and Ghazal formations varies from 8.4 to 28.0% (Table 6). Based on XRD analysis, the detrital clay minerals are mainly kaolinite and chlorite (Table 7). They were further analyzed using SEM technique (Fig. 10a–d). Figure 10 shows few amounts of stacked pseudo hexagonal plates of kaolinite partially filling a pore (b: E–F 5–6), considerable amount of pyrite, occurring within the pores (b: C–F 7–8; E–F 11–12: d: C–D 2–4 & E–H 11–13). Some authigenic kaolinites appear as pseudo-hexogonal plates arranged into an extended stacked form called verm (d): D–G 7–10 & A–B 11–12). Some rose-like shape of chlorite (d: A–D 3–7) was also detected. The kaolinite and chlorite can disaggregate into pore spaces and throats, thereby causing a decrease in porosity and permeability. The reservoir quality could be further enhanced by the dewatering process^38^. This assertion is supported by the absence or low smectite and illite minerals in all the studied samples yielding high secondary porosity (2.5% to 5.5%) with an average of 3.6%. Also, considerable amount of pyrite were observed within the pores (Fig. 10b: C–F 7–8; E–F 11–12), few quantities of iron oxides occurring between the detrital grains (Fig. 10b: B–C 6; F7) and minor amount of carbonate (Fig. 10b: F–G 8–9).

Quartz, pyrite, and siderites are the major cementing materials observed in Zarga and Ghazal sandstones. Quartz overgrowth is the major detracting factor reducing reservoir quality^8,39,40^. Although some mineral cements are in minor quantity, quartz overgrowth results in the reduction in pore spaces, which in turn causes a decrease in porosity and permeability^8^. Quartz overgrowth do not exceed 2.0%, occurring as nucleated cells around some of the quartz grains and grow into macro pores (e.g. Figs. 9c, 10c). It often results in reduction of macro porosity. Pyrite cement is in the range of 0.5% to 2.8% for most of the samples. Porosity and permeability are relatively low in the samples with higher pyrite concentration compared to ones with low pyrites^8^. Iron oxides also act as minor cementing agent filling pore spaces in the studied samples (Fig. 9a). Siderite, which is also considered as a cementing material occurs as pore-filling crystal between the grains (Fig. 9f).

Compaction is the degree to which sediments burial leads to porosity and permeability decrease with increasing depth due to effects of pressure from loading and cementation^8,40^. Most of the examined samples are poorly to moderately compacted. Sutured grain contact that usually reflects a higher degree of compaction was not observed on the coarse grain samples, but long grained concavo-convex contacts were noticed in some samples (e.g. Fig. 9c). Yet, the intergranular pore spaces left after compaction process are smaller in the fine-grained sediments (Fig. 9a). This affirms that compaction influences porosity and permeability. This is concordant with the findings above implying that sedimentology and grain textural features have direct effect on reservoir quality.

Conversely, the porosity and permeability of reservoirs can be further increased by dissolution of feldspars, micas, and carbonate cements^8,41^. Secondary porosities are primarily related to dissolution of feldspar minerals and calcite cement^41,42^. Secondary porosity is the most prevalent diagenetic process, depending on leaching of feldspar grains and dissolution of calcite cement^8,41^. This present study shows that K-feldspar has little degree of alteration and dissolution, ranging from partially dissolved (Fig. 9e) to relatively fresh unaltered grains (Fig. 9f). Alteration and dissolution are the two processes yielding secondary porosity in Zarga and Ghazal formations. These lead to kaolinization and quartz overgrowth in subsequent stages^8^. In the examined samples, kaolinite and quartz cement play less significant role on the reservoir quality as most of the studied samples have high porosity and permeability (Table 6), except high clayey samples (facies Sr). Chlorite which usually improves grain compaction resistance hinders quartz cementation, preserves the porosity and permeability as noticed in the studied samples^34,41^. Similarly, dissolution of mica resulted in secondary porosity. Minor amount of micas was observed in all the investigated samples (Table 6), which ascertains good quality reservoir. On the other hand, sediments having high mica content shows pressure solution compaction that damages intergranular porosity (Fig. 9a,b).

### Burial depth

**Figure 15:**
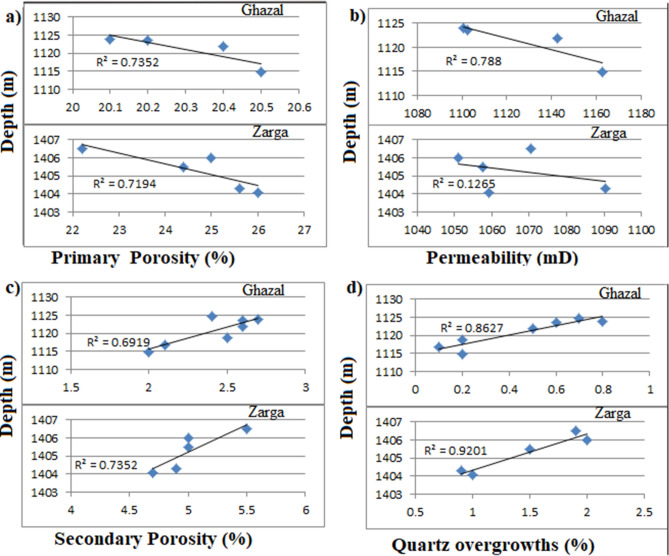
(**a**) Cross-plots of primary porosity and permeability values against burial depth, (**b**) showing that porosity and permeability decrease with increasing burial depth. (**c**) Secondary porosity displaying a positive trend with increasing burial depth. (**d**) Shows the amounts of quartz overgrowths increase with increasing burial depth.

The examined cores cover Zarga and Ghazal formations of the Keyi oilfield in the Northeastern Muglad Basin. Tables 2, 6 and 7 show the depth intervals of the core samples. The studied Ghazal formation (1114.85 m to 1124.70 m) samples are at relatively shallower burial depths compare to the Zarga formation samples (up to 1406.50 m). Zarga formation (Sm Sr, St & Sp facies) samples have slightly higher porosity and permeability than that of Ghazal formation (Table 6). The plots of primary porosity and permeability values against burial depth (Fig. 15a,b) fall within normal trend, implying that porosity and permeability decreases with increasing burial depth. On the other hand, secondary porosity of the analyzed facies (Sm Sr, St & Sp) displays a positive trend with the increasing burial depth (Fig. 15c). This is because as the burial depth increases, secondary porosity usually forms due to the dissolution of feldspar and other minerals as well as dewatering of sediments^34,38^. Moreover, dissolution of feldspar and other minerals will result in increasing the amounts of quartz overgrowths^8,38^. Figure 15d illustrates the increase in quartz overgrowths with increasing burial depth. This could be attributed to increase in temperature and pressure associated with higher depth^38^. Additionally, mechanical compaction acts as a key diagenetic process that leads to decrease in porosity and permeability with increasing burial depth^8,38^. The long and float grain contacts observed at the Zarga formation suggests a moderate compaction and porosity loss (Fig. 9c–f). Zarga formation reservoir quality is greatly influenced by secondary porosity. Meaning that, reservoir quality of the examined formations is also controlled by burial depth whereby primary porosity and permeability decreases with increasing burial depth due to compaction and cementation (quartz overgrowth).

### Reservoir quality

The obvious relationships between porosity and permeability as well as variation in depositional facies drive hydrocarbon migration and accumulation. These in turn depend on textural features, and diagenetic processes operating during or after time of deposition^41–43^. Hence, porosity and permeability are important for commercial hydrocarbon accumulation that and need to be quantitatively consistent to depict reservoir quality^8,41^.

**Figure 16:**
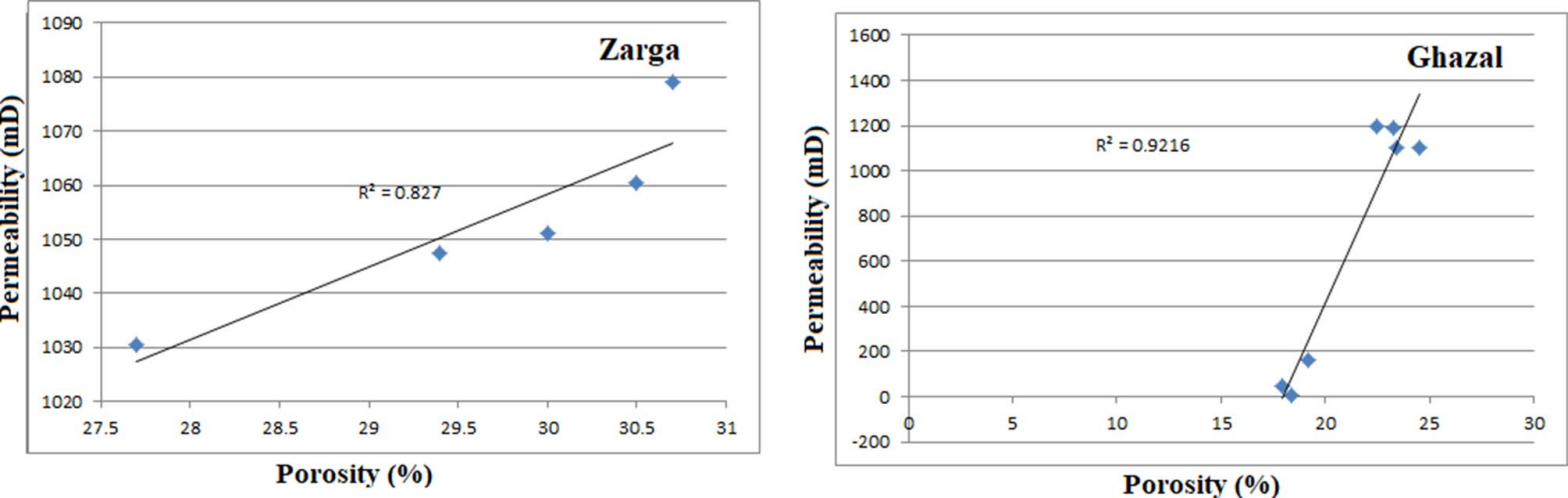
Cross-plot showing a positive correlation between porosity and permeability.

Even though the porosity of the examined samples is affected by presence of some mineral cements (pyrite, siderite, clays, iron oxides and quartz overgrowths), it is however, dominated by high intraparticle pores as well as large-scale secondary porosity through partial or complete dissolution of feldspar, mica, carbonate cements and clays. Overall porosity values of the Zarga formation samples range from 27.7 to 30.7%, while porosity of Ghazal formation ranges from 17.9% to 24.5 (Table 6). Average pore sizes are highly variable, ranging from 25 to 345 microns. Pore interconnectivity ranges from Fair to excellent (Table 6). Very weak calcite and pyrite cements possess relatively large pore sizes and good pore interconnectivity within the studied samples (Sm Sr, Sp & St facies). This can be a major contributing factor to the observed high permeability values in nearly all the examined samples ranging from 1051.09 mD to 1090.45 mD for the Zarga formation samples and 9.65 mD to 1196.71 mD for the Ghazal formation (Table 6). In this manner, samples (Sm Sr, Sp & St facies) from Zarga formation have better reservoir quality than Ghazal formation samples (Sr, Sp & St facies). Positive correlation between porosity and permeability proposes a close relationship, in which the high porosity corresponds to high permeability (Fig. 16).

The depositional environment is another factor controlling the shape, grain size of the sand body, sorting, structure, and diagenetic processes. These properties influence porosity and permeability of the sedimentary rocks^8,38,43^.

**Figure 17:**
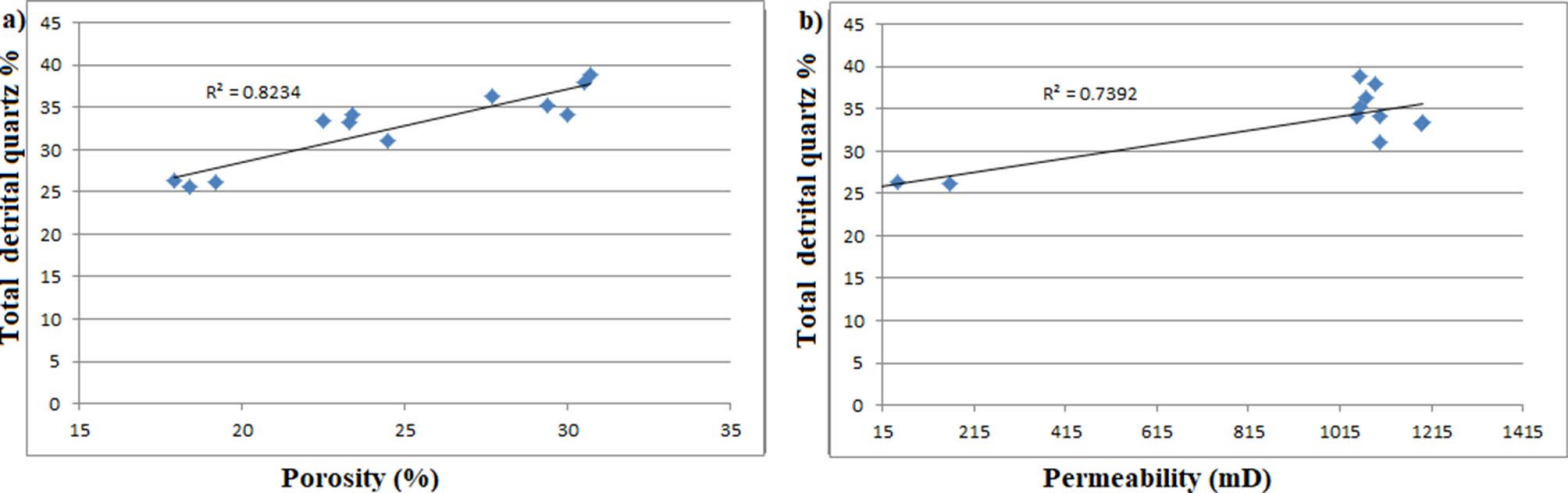
Cross-plot showing the relationship between detrital quartz content and porosity and permeability. The higher the detrital quartz content, the greater the porosity and permeability.

The sedimentary succession in core-1 and 2 of the Ghazal formation depicts a fluvial system followed by deltaic depositional environment, whereas the Zarga formation succession (core-3) is dominantly of deltaic settings (Table 1). This is consistence with the observed reservoir characteristics of the Zarga formation and the Ghazal formation depositional environments that contain a thick sandstone bed with less interbedded shale layers^1^. The reservoirs originated due to rapid sedimentation where large amounts of coarse-grained sediments were deposited with minor fine-grained rock (facies Sr), referred to as the Ghazal formation. These two types of sediments differ in porosity and permeability values. The coarser grained sediments possess porosity and permeability values ranging from 22.5 to 30.7% and from 1102.45 mD to 1196.71 mD, respectively, whereas the values in the fine grained sediments range from 17.9 to 19.2% porosity and 161.87 mD to 9.65 mD, respectively (Table 6). Furthermore, the coarse grained facies (e.g. Sm, Sp and St facies) are characterized by well sorted and sub-rounded to rounded grains. Generally, well sorted, sub-rounded to rounded sandstones with coarse grains indicates good reservoir quality as compared to fine grained sediments^8,44^. This signifies that, the Zarga formation sandstones have better fluid-flow propensities than the Ghazal formation reservoir sandstones. More so, structures and compositions of these two reservoir rocks also differs. For example, the detrital quartz content of the coarser grained sediments is higher ranging from 33.3 to 38.8% while the quartz in the fine-grained sediments is between 25.7 and 26.3%. The quartz is more mature and of greater compaction resistance, thereby maintaining primary porosity^34^ as observed in Fig. 17 illustrating greater porosity and permeability.

**Figure 18:**
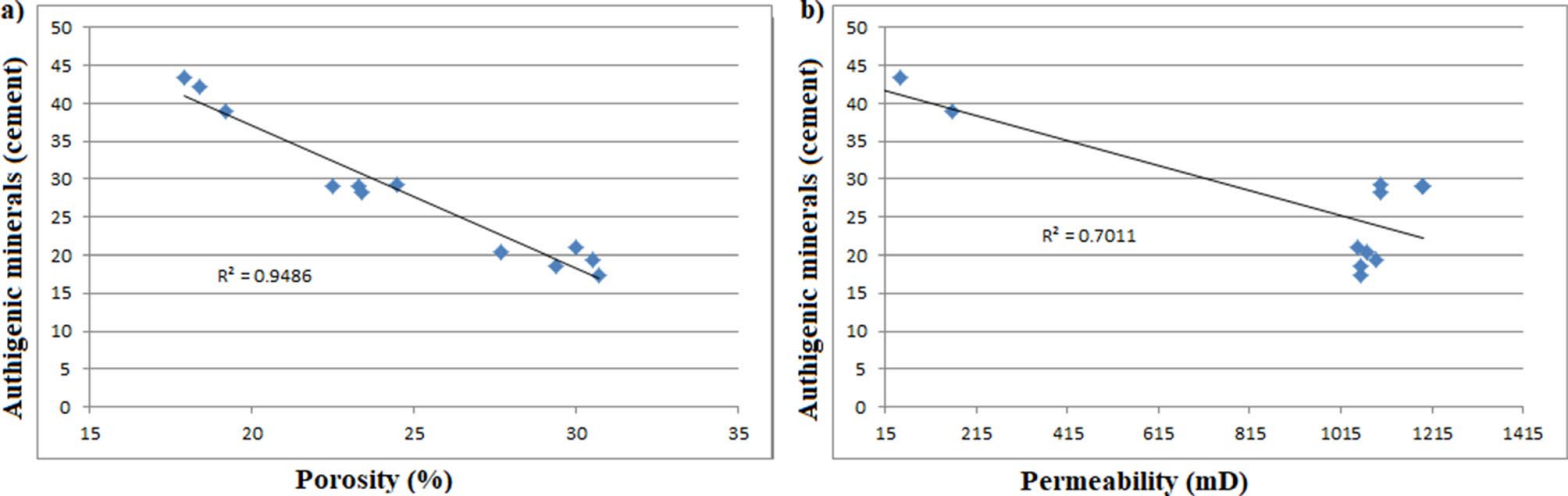
Cross-plots illustrating a good inverse correlation between authigenic mineral cements (including clay matrix) and porosity and permeability. This indicates that the cements are the major concern for the reservoir quality reduction.

Apart from depositional environment, the reservoir quality of the two formations is also controlled by diagenetic processes. The total clay content comprises of chlorite and kaolinite as pore filling cements. These clay minerals decrease porosity and permeability of the reservoir by blocking their pore spaces (Fig. 9b,d). Samples with higher clays tend to have lower non-micro porosity and permeability values (Table 6). Just as siderite, pyrite and iron oxides block pore throats thereby reducing the porosity and permeability of the samples. Figure 18 shows an increase cementation produces lower porosity and permeability. Quartz cementation and overgrowths (e.g. Figs. 9c,d, 10c) developed during late diagenesis as complete or incomplete rings around the quartz grains especially in moderately to deeply buried reservoirs^39,40^. Thus, increasing burial depth, compaction and quartz cementation tends to reduce porosity by transforming the grain contact from absolutely no contacts to point contacts or from point contacts to linear and concavo-convex contacts (e.g. Fig. 9c,d). Compaction and cementation are no doubt the major processes reducing reservoir quality. Just as the depositional environments of the examined sandstones control the shape, size, structure, and type of deposits, which in turn determine the reservoirs’ primary porosity and permeability. Therefore, compaction and cementation are the principal diagenetic processes reducing the reservoir quality steadily with increasing burial depth. Nevertheless, the reservoir quality is enhanced by dewatering and dissolution of feldspar, mica, and carbonate cements.

## Conclusion

Reservoir quality assessment of the Zarga and Ghazal formations (cores) in the Keyi Oilfields, northeastern Muglad Basin, Sudan was carried out using integrated analyses of sedimentary facies, depositional environments, and diagenesis. The studied reservoir intervals represent eight lithofacies facies: (i) ripple laminated sandstone and siltstone (SR), (ii) laminated to massive mudstone and shale (FCF/FM), (iii) massive sandstone (SM), (iv) trough cross-bedded sandstone (ST), (v) fine laminated mudstone and siltstone (FL), (vi) planar cross-bedded sandstone (SP), (vii) bioturbulent laminated sandstone (BS), and (viii) intraformational conglomerate (GM). These facies are generally medium to coarse-grained sandstone, except the rippled-laminated sandstone and siltstone facies (SR), which is dominantly characterized by fine-grained sediments.

The reservoir sandstone intervals are texturally immature and associated with high sedimentation rate occasioned by high-energy fluvial channels dominated by bedload transport as evidenced by the preponderation of high quartz and feldspars contents and general fining upward stacking pattern in the cores. The integration of petrophysical and pore-imaging datasets from the studied formations shows that the best reservoir quality in the field is linked with coarse-grained, moderately sorted, and high-energy channel sandstones.

The observed low porosity and permeability in these facies resulted from the abundance of argillaceous content (reservoir fines) and the extensive carbonate and clay cement types. Both the primary and secondary poro-perm values are controlled by burial depth in the Keyi Oilfield, Sudan. The former has an inverse relationship with the burial depth, whereas the latter has a direct correlation with the burial depth. Thus, both primary porosity-reducing and secondary porosity enhancement processes simultaneously evolved in the formations. Diagenetic effects such as compaction, precipitation of authigenic kaolinite and iron oxides, sideritic and pyritic cementations, and quartz overgrowths are the important reducers of depositional porosity and permeability of the Zarga and Ghazal formations reservoir units. Conversely, grain dissolution is the paramount secondary porosity-enhancing process in the studied reservoirs.
